# Cold Spray: Over 30 Years of Development Toward a Hot Future

**DOI:** 10.1007/s11666-022-01366-4

**Published:** 2022-05-02

**Authors:** D. Guo, M. Kazasidis, A. Hawkins, N. Fan, Z. Leclerc, D. MacDonald, A. Nastic, R. Nikbakht, R. Ortiz-Fernandez, S. Rahmati, M. Razavipour, P. Richer, S. Yin, R. Lupoi, B. Jodoin

**Affiliations:** 1 Cold Spray Laboratory, University of Ottawa, Ottawa, ON Canada; 2 Trinity College Dublin, The University of Dublin, Department of Mechanical, Manufacturing & Biomedical Engineering, Parsons Building, Dublin, Ireland

**Keywords:** Cold spray, Hype cycle, Powder deposition, Innovation

## Abstract

Cold Spray (CS) is a deposition process, part of the thermal spray family. In this method, powder particles are accelerated at supersonic speed within a nozzle; impacts against a substrate material triggers a complex process, ultimately leading to consolidation and bonding. CS, in its modern form, has been around for approximately 30 years and has undergone through exciting and unprecedented developmental steps. In this article, we have summarized the key inventions and sub-inventions which pioneered the innovation aspect to the process that is known today, and the key breakthroughs related to the processing of materials CS is currently mastering. CS has not followed a liner path since its invention, but an evolution more similar to a hype cycle: high initial growth of expectations, followed by a decrease in interest and a renewed thrust pushed by a number of demonstrated industrial applications. The process interest is expected to continue (gently) to grow, alongside with further development of equipment and feedstock materials specific for CS processing. A number of current applications have been identified the areas that the process is likely to be the most disruptive in the medium-long term future have been laid down.

## Introduction

Often referred to as the “latest” thermal spray process, cold spray has been around for over 30 years. It has indisputably attracted the attention of many researchers, scientists, and industrials very quickly due to the apparent simplicity of the process and the many possibilities that it was offering. This paper provides an overview of the evolution of the process, illustrating that while the underlying physics is more complex and fascinating than one could have foreseen, the past 30 years have been a developmental roller coaster allowing the process to mature and find its own market niche that points to an even brighter future. The first part of the paper provides an historical technical review of the process origin and developments, while the second part tentatively offers potential future niche applications.

**Fig. 1:**
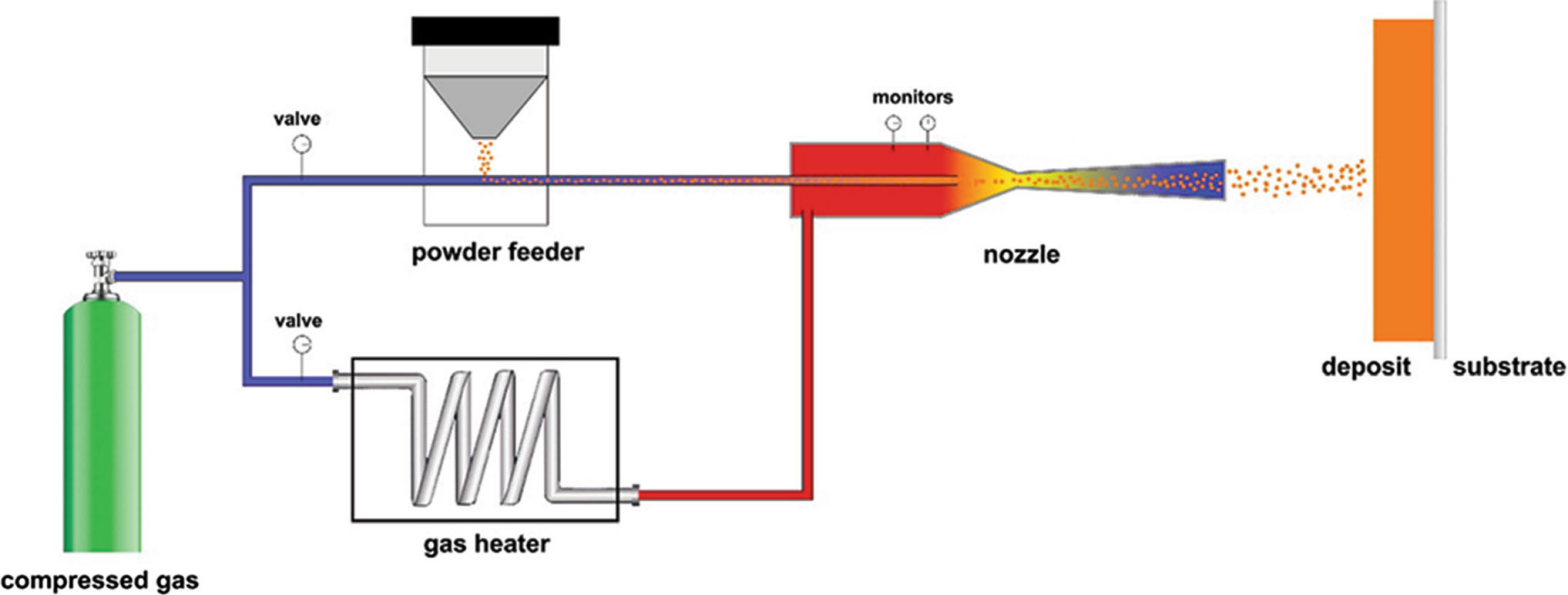
Working mechanism of a typical cold spray system

The cold spray (CS) solid-state material deposition process was developed in the 1980s at the Institute for Theoretical and Applied Mechanics of Russia (Ref 1, 2). In CS, compressed gases (air, nitrogen, helium) at temperatures up to 1000 °C are used as propellants to accelerate metallic and/or ceramic feedstock powder to a high velocity (300 to 1200 m/s) in a convergent-divergent (de Laval) nozzle. Upon impact onto the substrate surface, the powder particles experience severe plastic deformation and adhere to the substrate or previously deposited particles to form coatings or bulk deposits. Figure 1 shows schematically the working mechanism of the CS process. Operating parameters controlling the CS process include gas parameters (pressure, temperature, and type), powder feed rate, nozzle internal geometry, scanning strategy (scanning step and pattern, nozzle traverse speed). CS was initially applied as a coating technology for corrosion, wear, oxidization, and thermal protection. As opposed to other thermal spray processes, the formation of a CS deposit relies mainly on the particle kinetic energy prior to impact rather than thermal energy. CS particles remain in the solid state during the entire deposition process, and adhesion/cohesion of deposited particles is achieved through local metallurgical bonding or mechanical anchoring. As such, the defects commonly encountered in high-temperature deposition processes such as oxidation, tensile residual stresses, and phase transformation can be prevented (Ref 3, 4).

In recent years, the rapid development of modern manufacturing technologies resulted in the expansion of the CS application window from a coating to an additive manufacturing (AM) process. In AM, digital files of components are directly translated into net or near-net shape through a layer-by-layer construction process. As part of Industry 4.0, AM has become a major technology worldwide. As a new member of the AM family, CS can fabricate free-standing metal/cermet components and also restore damaged metallic components (Ref 5-7). Compared to commonly used fusion-based AM technologies (such as Powder Bed Fusion and Directed Energy Deposition), CS has unique advantages such as shorter production times (high deposition rate), unlimited product size (no built-tray required), reduced thermal effects, and high adaptability to different materials (Ref 8, 9). CS is particularly suitable for fabricating components made of high-reflectivity metals that are typically difficult to manufacture using laser-based AM processes. It is, therefore, regarded as the ‘Next Generation Additive Manufacturing’ (Ref 5). However, the current challenge of cold spray additive manufacturing (CSAM) is the control of the properties of the as-made deposits or parts. This is why in the past decades, a number of effective pre-processing, in-process, and post-processing technologies have been developed in an attempt to address this problem.

After 30 years of development, the CS technology has experienced several scientific and technological breakthroughs such as new bonding mechanisms, in-situ process visualization, new materials, new concepts, new CS apparatus configurations, new pre-, in-, and post process treatment approaches, and new applications. All these efforts have led to a modern and mature CS technology, and its fields of application are increasingly wider. In this paper, the development of the CS process over the past 30 years is systematically reviewed. The paper starts from a comprehensive introduction on the history of cold spray technology, and then summarizes the key breakthroughs that significantly improve our understanding on CS and contribute to the upgrade this technology. After that, the evolution of technological innovations of CS from conception to maturity is reviewed using a “hype cycle model” especially adapted for CS. Thereafter, the focus is switched to the summarization of various CS coatings and their applications in industry. At the end, a conclusion drawn from the paper and the future perspective of CS technology are provided. It is expected that this review can provide guidelines for people who are and will be working with CS to well understand the innovative CS technology.

## The Cold Spray Journey

### Technological History and Improvements

Despite the fact that CS is still considered to be a novel thermal spray technique, its concept and main principles have been carved out by several pioneering minds for more than one century.

#### Early Concepts

**Fig. 2:**
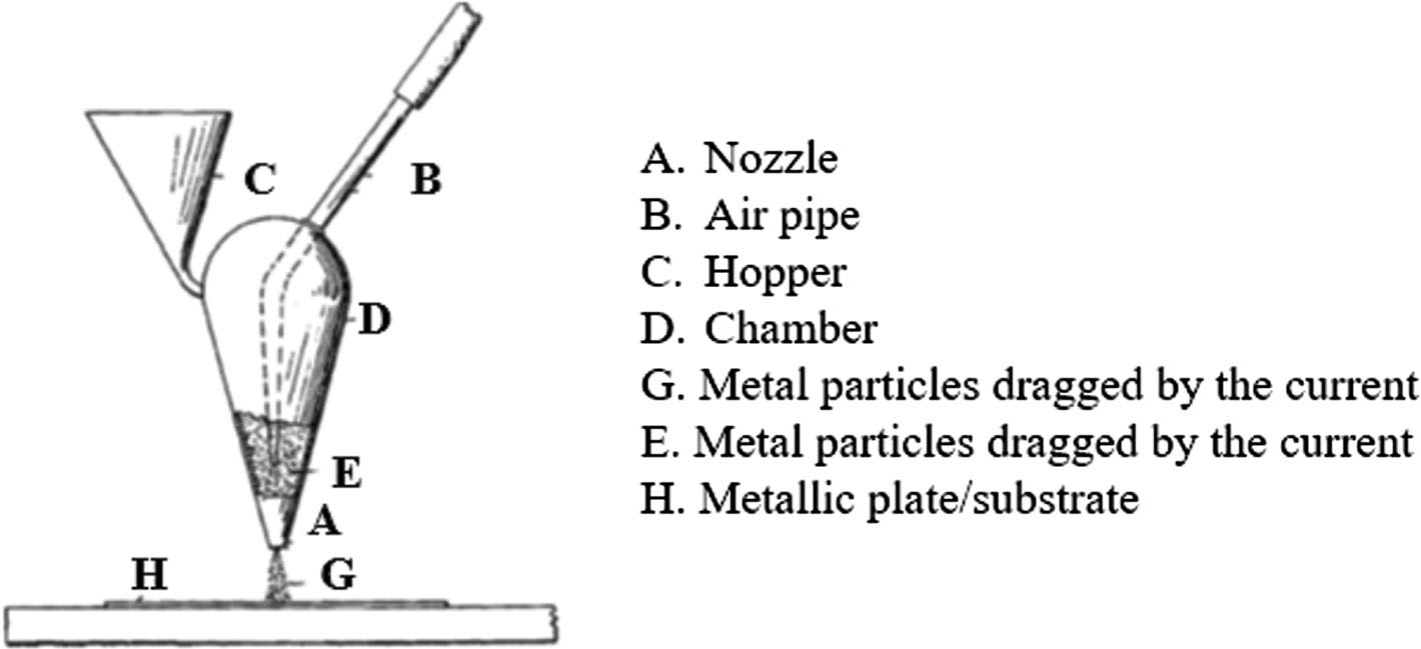
Apparatus for impacting one metal upon another modified by (Ref 10)

In 1898, Thurston (Ref 10) filed a US patent (issued two years later) that can be considered as a precursor of the modern CS machines (see Fig. 2). The patent claimed the ability to deposit copper and aluminum coatings upon copper and steel substrates. It also stated that substrate preheating may facilitate the consolidation process, implying knowledge that sufficient plastic deformation can be triggered by increased surface temperatures. The absence of external regulation of powder feeding and limitation to subsonic flow regime inside the nozzle (Ref 11) suggest limiting capabilities of the device. Thurston also reported that superheated steam could be used to accelerate the particles, a detail that was omitted a few years later in a follow-up patent (Ref 12) where the inventor underpined the importance of oxidized-free surfaces for the successful deposition of a coherent coating.

**Fig. 3:**
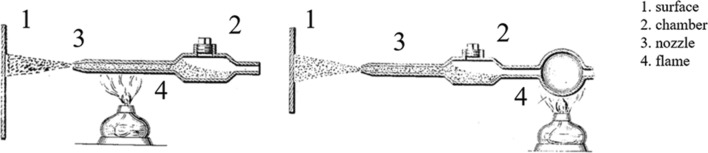
Indicative drawings of Schoop's invention, modified by (Ref 14)

In 1909, Dr. M.U. Schoop (Ref 13), the father of thermal spray, investigated the adherence of low melting point materials (tin and lead) in granular form when fired against a wall using a small cannon. Α few years later (1915), he filed a patent describing a “Method of Plating or Coating with Metallic Coatings” (Ref 14), which included various alternatives to consolidate solid particle feedstock dragged by a heated propellant gas onto a substrate with high impact velocity. An indicative setup of his invention is presented in Fig. 3.

Schoop highlighted the versatility of this technique as solid form (particles) instead of molten material usually used up to that point constituted the feedstock, preventing thermal damages to substrates vulnerable to excessive heat. He also reported (Ref 14) the use of powder heating to facilitate the deposition of the solid particles projected with high kinetic energy. Despite the fact that modern CS operates according to very similar concepts, it is not clear whether the powder remained in solid state upon impact. Schoop mentioned that the particles and the substrate “weld together” while he did not use a converging-diverging nozzle (see Fig. 3) to accelerate particles up to a critical velocity. This suggests that the bonding mechanism most likely involved the melting or partial melting of the particles.

**Fig. 4:**
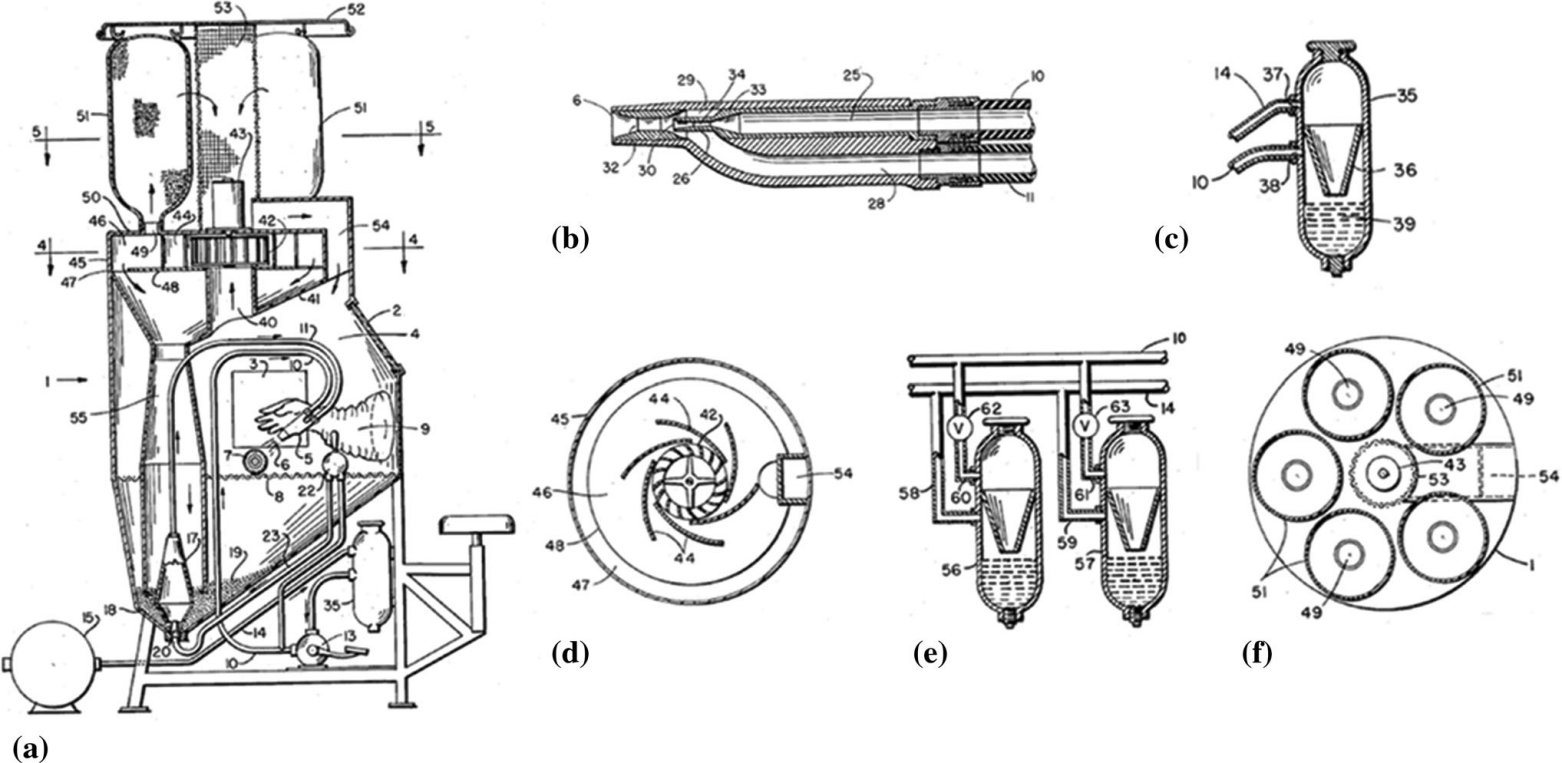
Device for treating the surface of a workpiece as modified by (Ref 15). Sectional views of the a) device, b) nozzle, c) liquid container, d) dust separator, e) arrangement of fluid containers, f) filter bags and housing inlet

Rocheville (Ref 15) described a device for treating the surface of a workpiece in a 1958 patent application (issued in 1963). The overall cross-section of the device is presented in Fig. 4a. Figure 4b shows the internal details of the nozzle. The machine also had the capability of ejecting liquid binder stored in a closed container (Fig. 4c) that was combined in some cases with dry lubricant material to form a coating on the workpiece surface. There was also an option for use of mixtures of several liquids stored in appropriate containers (Fig. 4d). The powder (dust) that did not consolidate on the workpiece could recirculate after being separated in a dust separator (Fig. 4e) while the air could be filtered after passing through filter bags (Fig. 4f).

**Fig. 5:**
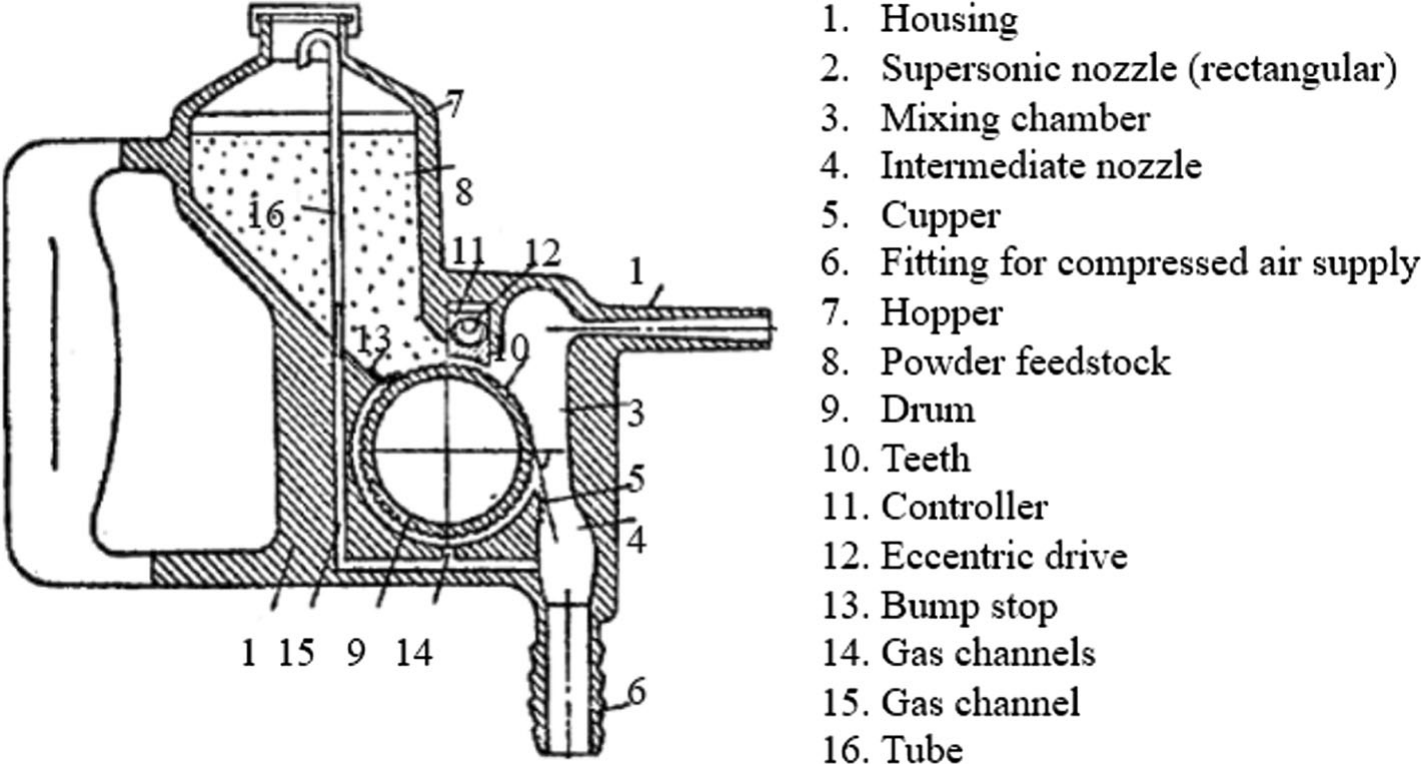
The first apparatus (Ref 21) for the fabrication of cold spray coatings (granted in 1991, Soviet Union)

This device was mentioned to operate with air and using a convergent-divergent nozzle for particle acceleration at supersonic velocities. It was able to clean surfaces, relax residual stresses or create extremely thin coatings. It was likely working similarly to abrasive blasting systems, being able to remove layers or embed particles monolayers on the substrate surface to form thin films, with mechanical interlocking to be the primary deposition mechanism. This is clear as the inventor reports that the thickness of the layer is expected to be very thin (in the order of 2.5 μm) and that the particles can adhere to the substrate but to build up a coating with layer-by-layer strategy despite the supersonic velocity. There are two main factors that might contributed to the limited capabilities of Rocheville’s invention. Firstly, the critical velocity to deposit particle upon particle could not be reached by that time. In other words, the machine could not work inside the window of deposition, a theory that has been developed in recent years (Ref 16). Secondly, the contemporary metallic powders couldn’t undergo significant plastic deformation that is a prerequisite for building thick coatings (Fig. 5).

#### Modern Era in Soviet Union and Russia

The modern era of CS commenced at the Institute of Theoretical and Applied Mechanics of the Siberian Branch of the Russian Academy of Science (ITAM SB RAS) in Novosibirsk. During late 1970s, a Soviet research team led by Papyrin and Alkhimov investigated supersonic two-phase flows composed of air and several metallic, organic, and biological micro-particles (Ref 17). It was reported (Ref 18) that motion of particles in the stagnation region can lead to surface erosion, but also to consolidation and the formation of coatings that would stay attached to a substrate material. As mentioned by Papyrin et al. (Ref 19), the velocity-driven consolidation mechanism of CS was revealed when they noticed the consolidation of aluminum particles with a velocity of 400-450 m/s on a cylindrical body at a stagnation temperature of 7 °C (280 K).

Understanding the importance of these early-stage observations, Alkhimov et al. filed (Ref 20, 21) the first patents in 1986 (issued in1991). In the first patent (Ref 20), the authors referred to an unheated gas flow of air, argon, helium, or their mixture that is capable of dragging particles with diameters ranging from 1 to 200 μm and form solid coatings. The second patent (Ref 21) reported analytically the parts of the device used for the fabrication of coatings. It also claimed that particle velocities could exceed 1000 m/s and that the turbulence of small particles (1-20 μm) into the gas stream can be reduced. In 1994, Alkhimov et al. (Ref 22) reported that they were able to deposit apart from aluminum, a wide variety of metals including Zn, Cu, Fe, Ti, V, Co, Ni, and Sn. The properties of CS coatings were found to be dependent on the operating conditions, with porosity in the order of 1%, adhesion strength from 30 to 80 MPa, and thicknesses in the range between 10 and 10^4^ microns.

Following the early discoveries in ITAM, the Obninsk Center for Powder Spraying (OCPS) and the Aviation Institute (Ref 11) pioneered further development of the CS technique. OCPS (Ref 23) started to produce, sell, and support DYMET commercial coating equipment in 1992. This technological progress was accompanied by several patents. Russian inventors patented 14 inventions prior to 1995, before CS started to gain interest outside Russia (Ref 11). Patents rose to 37 until 2003 when global research on this field expanded rapidly. The main advances during the years 1986-2003 pertained to the powder feedstock, the individual components of the CS device, as well as the way the full machine is assembled, integrated and controlled in a functional apparatus, and can be classified into the following categories:

### Feedstock Powder

The feedstock used for cold spray is normally gas-atomized or plasma-atomized spherical metal and pre-alloyed powders (Ref 24). Occasionally, water-atomized powders with irregular shape can also be used for cold spray, but irregular powders lead to lower strength and properties as compared to their spherical counterparts. Cold spray also has strict requirement on powder size and size range. In general, powders with a size ranging between 10 and 100 μm in diameter are deemed suitable for cold spray deposition. The powders with diameters greater than 100 μm or lower than 10 μm are difficult to accelerate by the driving as and thus usually fail the deposition (Ref 25). However, it is worth noting that the most frequently used size-range is 20 - 60 μm for most metals and alloys. For those having relatively low density such as aluminum and zinc, the upper limit of the size-range can be pushed to 100 μm. In terms of powder materials, cold spray has preference on the materials with high ductility and low strength (e.g., copper and aluminum). This is because of the nature of cold spray that requires extensive plastic deformation of the powder materials upon impact to form coatings or deposits. The deposition of high-strength materials (e.g, Inconel and steel) requires high gas parameters or other pretreatment (e.g., powder preheating and powder annealing) or in-process treatment (e.g., laser assistant and in-situ peening).

Mixtures of ductile and brittle feedstock powders were used by Buzdygar et al. (Ref 26) to increase the density and hardness of CS coatings, attributed to the hammering mechanism when ceramic particles impinge on the as-deposited metal particles. Similarly, Kashirin et al. (Ref 27) used binary mixtures of metallic feedstock powders and hard spherical particles with diameters exceeding 30 μm to achieve a similar result. The same researcher (Ref 28) applied abrasive particles with diameters in the range of 30 to 300 μm to prepare the surface to be coated in a way similar to abrasive blasting, but using the CS machine instead. This ensured the cleanliness of the coating before the final spray of the metal feedstock powders while a major beneficial aspect of this method is the reduction of the fabrication time as the surface preparation and the coating deposition stages take place in one step.

### Powder Feeding

Buzdygar et al. (Ref 29) experimented with separate powders simultaneously fed into the system. They managed to improve the deposition efficiency and avoid the erosion of the nozzle inner surface by feeding powders into different locations of the nozzle. More specifically, metallic powder was supplied into the converging part of the nozzle, while ceramic powder was supplied into the diverging part. Dikun (Ref 30) used different powders fed individually into the system from separated gas lines to fabricate composite metallic coatings. The powders accelerated into the elongated part of the nozzle were subsequently blended by the gas stream. The dynamic action of the gas initiated an exothermic reaction as the powders entered the regime of high temperature self-propagating synthesis. Shkodkin (Ref 31) designed and patented an invention that used a heat exchanger to dissipate heat from the nozzle walls and preheat the powder before entering the nozzle divergent part.

### Nozzle Design

**Fig. 6:**
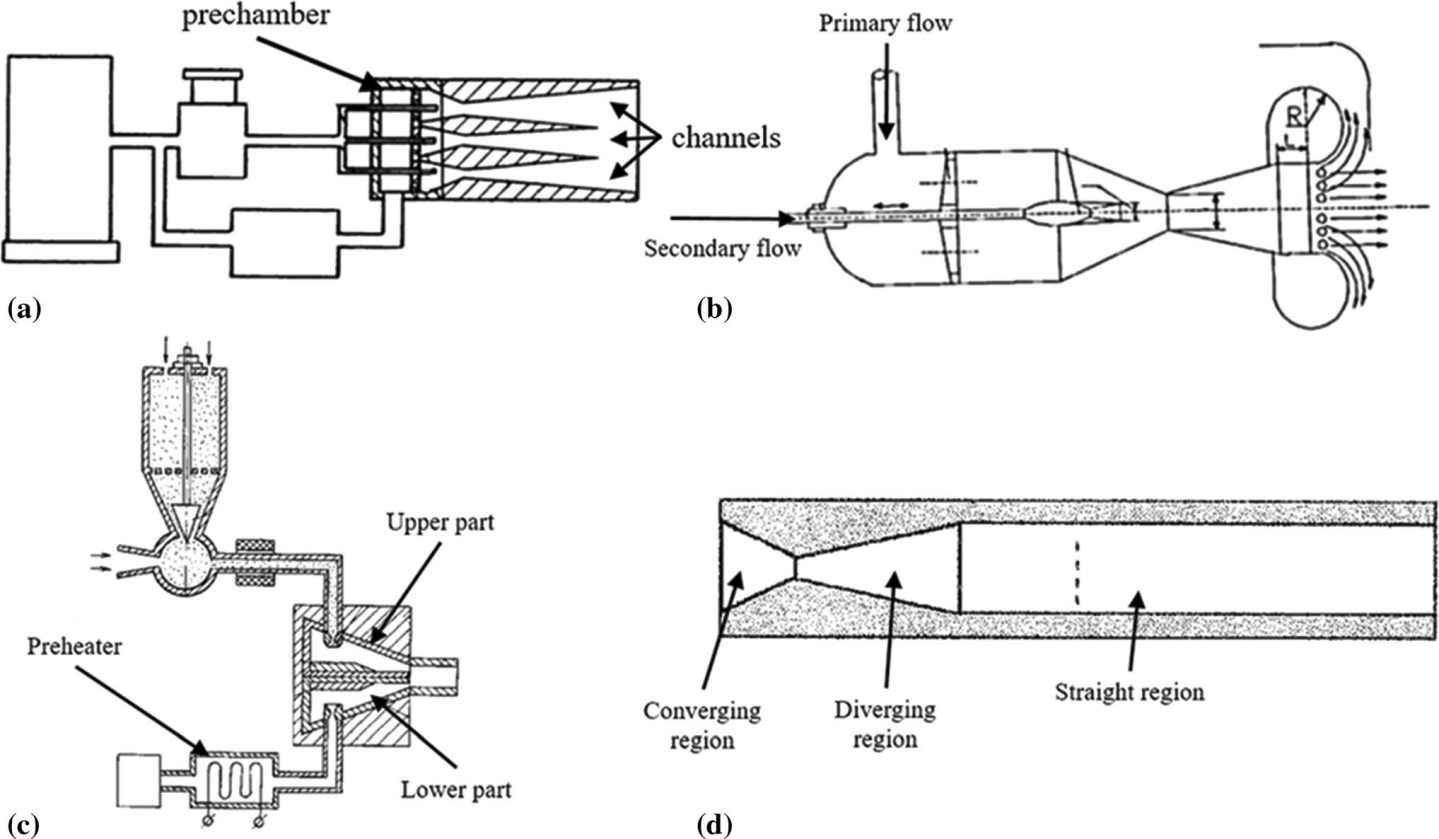
Various nozzle designs as proposed by (a) Alkhimov (Ref 29), (b) Dikun (Ref 30), (c) Krysa (Ref 31), and (d) Kashirin (Ref 32)

Alkhimov et al. (Ref 32) designed a nozzle configuration to increase the productivity of the spraying process, as well as to fabricate coatings of uniform thickness. In his design, the powder was fed in a pre-chamber and mixed with the preheated gas. Individual pneumatic channels (3) with converging-diverging profiles accelerated the particles into a common output channel before they impinge on the substrate (Fig. 6a). In this way, the sprayed projected area was significantly larger compared to using a single nozzle. Dikun (Ref 33) used a nozzle design that could accelerate the particles by a gas flow prior to their injection in the main stream (Fig. 6b). Another nozzle introduced by Krysa et al. (Ref 34) consisted of two parts (upper and lower) (Fig. 6c). Two separate lines fed the nozzle parts: unheated gas with powder were fed into the upper part, and preheated gas was fed in the lower part. The two flows superimposed in the elongated-straight part of the nozzle. In 2003, Kashirin at al. (Ref 35) used a nozzle that reduced the speed of the gas flow after reaching supersonic region and before impinging on the substrate. Enhanced deposition efficiency could be achieved at speeds slower than the speed of sound, as the particles could remain at high temperatures and consolidate easier due to thermal softening.

### Gas Heating

**Fig. 7:**
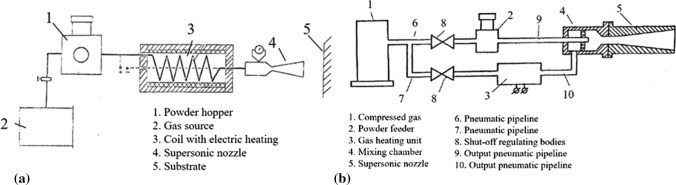
Early-stage patents for cold spray with gas heating: (a) Gas powder supplied from the same line, (b) Powder and gas supplied from separate lines

A preheated gas chamber was firstly introduced in 1987 by Alkhimov et al. (Ref 36), where an approximate temperature of 0.25-0.65 of the powder melting temperature was reached. In this invention, the powder was fed into the main line with the gas, heated into the feeder and finally projected onto the substrate as presented in Fig. 7a. The same research team (Ref 37) redesigned their original machine providing separate lines for conducting the preheating gas and powder into the mixing chamber before accelerated through the nozzle (see Fig. 7b).

Alkhimov et al. (Ref 38) used binary mixtures of helium and air as propellant gas, with gas preheating to a maximum temperature of 400 °C. They estimated that the particle velocity lay within the range of 300 to 1200 m/s. Finally, Dikun et al. (Ref 39) used gas preheating to trigger chemical reactions in certain materials. A composite mixture of Zn ad Cu powders was sprayed to produce a brass coating. In this way, they managed to increase the hardness due to the presence of γ phase, without simultaneous embrittlement of the coating.

#### Worldwide Process Development

CS developments outside Russia started to take place in the mid-90s when the know-how of the technique was transferred to USA. More specifically, in 1994 Papyrin, started to conduct CS research at the National Center for Manufacturing Sciences (NCMS), in Michigan. Several companies^1^ constituted a consortium that supported his research team aiming to produce high-quality industrial coatings and commercialize the CS technique (Ref 40). In 1995, McCune, Papyrin et al. presented (Ref 41) the principles of the technique in an open audience, in the framework of the 8th National Thermal Spray Conference in Houston. In the following years, General Motors, ASB Industries, and Sandia National Laboratories built CS equipment, while incorporating CS into their R&D departments (Ref 42). Sandia National Laboratories published various works (Ref 42) on the economics, materials, modeling (Ref 43), and bonding mechanisms of the CS process. Meanwhile, Canada also attracted Russian researchers from Obninsk Center for Powder Spraying to the University of Windsor (Ontario). Their research mainly focused on Low-Pressure Cold Spray (LPCS) that was previously introduced by Kashirin et al. in USA (Ref 44). LPCS originally differed from high pressure cold spray (HPCS) as it uses approximately three times lower gas pressure (∼9 bar) and compressed air as propellant gas, offering high flexibility and lower equipment cost at the expense of an inferior coating microstructure (Ref 45). Modern LPCS systems attribute their name mainly to the pressure level at the location of powder which is low as the downstream powder injection is followed (Ref 46).

Under these advances, Centerline (Windsor) Ltd via its Supersonic Spray Technology (SST) division (Ref 11), started to produce and supply commercial CS systems from Canada to the North American market. USA pioneered CS developments during the years 2000-2007 with General Motors to be the leading institution (Ref 11); other automotive and aerospace industries were the main beneficiaries with the majority of end users. The invention and application of gas recovery systems (Ref 47) were aimed at reducing the process costs when helium is used as propellant gas. Moreover, despite the fact that CS was (and still is) synonymous to supersonic flow, slower (sonic and subsonic) flows were found to also be efficient to consolidate particles when powder preheating compensated for the reduced kinetic energy. This CS variant was patented and developed by Tapphorn and Gabel (Ref 48) under the name “kinetic metallization”. Cold spray operation under vacuum conditions firstly appeared in early ‘00s (Ref 49) with the main advantages to be the hindering of the bow shock effect, the deposition of small particle sizes and the achievement of high velocities with lower gas pressures. The particular CS technique was also mentioned as aerosol deposition method (Ref 50)

The deposition was further improved with the assistance of laser processing used to increase the temperature of the substrate and/or feedstock. Bray et al. (Ref 51) presented Laser-assisted material spray (LAMS) in 2006 for Al and Al-Ti feedstock powders on carbon steel substrate, presenting enhanced deposition despite the lower particle velocities. LAMS was later renamed LACS (Laser-assisted cold spray); the process can be further classified/categorized based on the arrangement of the laser beam and the CS nozzle (Ref 52). In one case, the laser beam precedes the CS jet by a few milliseconds to thermally soften the substrate, while in the other case, it is concentrically coupled with the CS jet to thermally process both the injected particles and the substrate. In a third case, the laser beam followed the CS nozzle in order to eliminate porosity via coating fusion. It should be mentioned that in the latter LACS variant (patented in 2006 (Ref 53), laser was used as an efficient in-situ thermal treatment method to eliminate already deposited cold sprayed layers, rather than enhance the deposition efficiency while spraying.

Another CS variant entitled pulsed-gas dynamic spraying (PGDS) was investigated (Ref 54) at the University of Ottawa (Canada). The research was based on an earlier invention patented by Dikun et al. (Ref 55). The particular technique is based on the intermittent use of a valve to drive a periodic shock wave created by a shock generator at a frequency between 2 and 50 Hz with the use of a propellant gas. The shock wave increases the temperature of the feedstock while creating an instant supersonic flow that drags and deposits the particles on the substrate. In every pulse, a definite amount of powder consolidates into a coating. The main advantages of the technique are the reduction of gas consumption and the increase in the energy efficiency. This technique was particularly applied for the spraying of Metal Matrix Composites (MMC) (Ref 56, 57).

CS was recognized as an emerging technique in Europe in early 2000s, where the relevant research started to flourish in Germany (Ref 58). The principal research was carried out at the University of the Federal Armed Forces in Hamburg with a number of landmark investigations that pertain to the bonding mechanism (Ref 59, 60), the microstructure of the coatings (Ref 61), and their electrical conductivity (Ref 62). The partnership between Linde R&D and CGT Technologies resulted in the development of the Kinetics 3000 CS system. CS applications expanded to coating and repair of medical engineering components and worn chills used in casting (Ref 58). In the early 2000s, CS started to emerge in Asia with China, Japan, and Korea being the main players in terms of research and commercialization. Contemporary CS developments aim to make the technique greener, more agile, economic, and efficient. Apart from gas recycling, powder recycling is currently considered an attractive innovation as it has recently shown significant potential (Ref 63). Contemporary CS systems are well automated (Ref 64), attain power consumption up to 70 kW, gas pressure up to 70 bar, and gas preheating temperatures up to 1100 °C depending on whether they use downstream or upstream injection approaches (Ref 65). Research focuses on the expansion of materials that can be atomized and sprayed. Such materials include high entropy alloys (Ref 66), superalloys (Ref 67), composites (Ref 68), stainless steel alloys (Ref 69), shape memory alloys (Ref 70), and metallic glasses (Ref 71).

### Key Breakthroughs

This section includes what is believed to have been the key breakthroughs for modern CS since its first appearance. In drawing this list, extensive judgment was applied in relation to what topic to bring forward, resulting in a selection characterized by a high level of novelty and scientific discovery (with respect to the timeframe), potential for further development, potential for industry applications, and uniqueness versus other spray processes. It must be pointed out that the number of citations in the papers consulted was not a metric considered in the selection process. The main advances of modern CS can be summarized as follows:

- Deposition window concept: this is possibly the most important scientific discovery in the field. The concept and identification of a deposition window have led to a clear process differentiation in terms of what materials can be used in this process. Deposition occurs when the particle impact velocity is located within a specific range, the latter being material-dependent.
- Polymer surfaces: the deposition of metallic materials over polymers can have several applications such as in aerospace and biomedical fields. It is, however, a difficult thing to achieve with common thermal spray methods. CS was able to produce, with surprisingly relative ease, metallic coatings over polymer surfaces without any distortion.
- Pulsed cold spray, Laser-Assisted cold spray and Micro-cold spray: these process variations are important because they are the first of their kind, and they attempted to bring CS closer to a process that is industrially attractive by the elimination of costly helium and the introduction of alternative innovations (pulsed and laser variation). The micro cold spray, on the other hand, represents an interesting attempt to turn the process into a line-printing precision technique that has many commercial applications.
- The tamping effect: this mechanism is at the core of the functionality of low-pressure CS. Impact “tamping” of a specific powder material added to the feedstock (typically a ceramic) can promote deposition under impact velocities below the critical value.
- Intermetallics in CS: this is a very interesting discovery that caused controversy for several years until it was fully proven. High-speed particle impact can indeed promote, under certain conditions, a metallurgical type of bonding with transition phases from the substrate to coating as an example. The most direct effect of this may be an increase in coating bond strength.
- High-speed videos of particle impacts: this is a very recent development, not easy to be achieved considering the extreme conditions in CS but quite impressive in terms of images sequence. It has opened a new branch of research in the area and will be an important contributor to fundamental knowledge.
- CS coatings can have oxides: this is at the least as striking as the intermetallics breakthrough. Oxides in CS shall not really form being a solid-state process, but this is most definitely confirmed not to be the case as there is indeed an activation mechanism.
- CS additive manufacturing: arguably the breakthrough with the most valuable industrial potential when looking at the future. It has been explored since the past decade, but only recently CSAM mechanical properties are starting to compare very well versus the bulk material counterpart.

#### Bonding Mechanisms and Deposition Window Concept

**Fig. 8:**
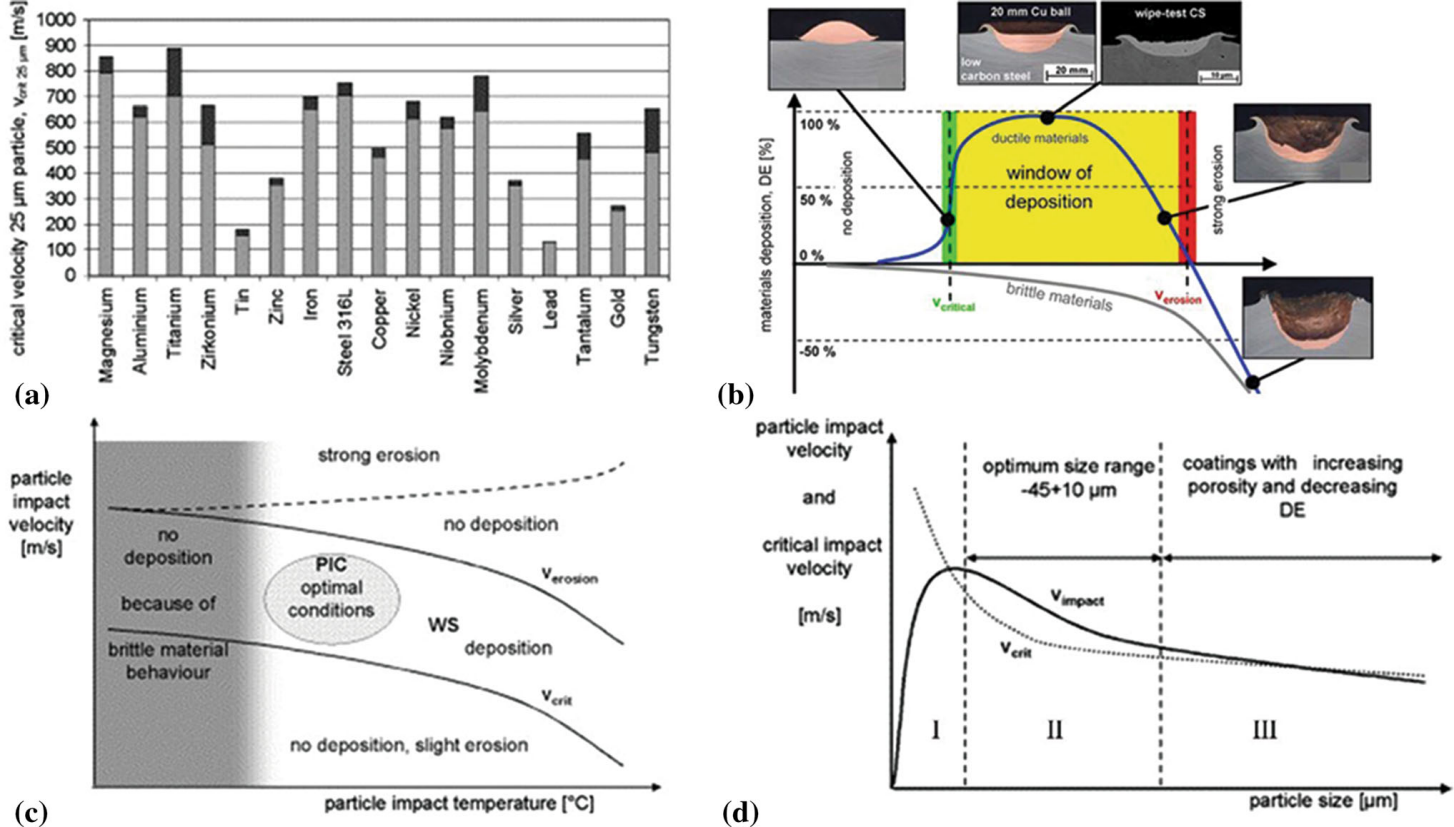
(a) Critical velocity of various materials; (b) Correlation between particle velocity, deposition efficiency and impact effects for a constant impact temperature, successful bonding occurs at deposition window; (c) Critical velocity and impact velocity over particle impact temperature; (d) Critical velocity and impact velocity over particle size (Ref 16, 75)

In the early ‘00s, Assadi et al. (Ref 59) reported that solid bonding at the particle/substrate or particle/particle interface results from adiabatic shear instabilities when the particle impact velocity exceeds a so-called critical velocity as reported by several researchers (Ref 59, 62, 72). Due to the high nonuniformity of the strain and temperature upon impact, the particle is suggested to develop only localized bonds at a fraction of the interacting contour. The adiabatic shear instabilities have been reported to be accompanied by the injection of out-flowing jets of plastically deformed material that contribute to the production of clean contact surfaces (Ref 73). Nevertheless, recent investigations conducted by Hassani et al. (Ref 74) demonstrated that adiabatic softening and adiabatic shear instability are not prerequisites for the formation of hydrodynamic jetting. Figure 8a shows the critical velocities of various metallic particles with a diameter of 25 μm. When the particle impact velocity largely exceeds the critical velocity, substrate erosion occurs instead of deposition (Ref 16). The interval between critical velocity and erosion velocity is defined as the deposition window, for which high material deposition efficiency is observed, as shown in Fig. 8b. The critical velocity mainly depends on the intrinsic properties of particles (e.g., material properties, morphology, and size) and particle impact temperature. Figure 8c shows the relationship between critical velocity and erosion velocity versus particle impact temperature. Some materials (e.g., tantalum, niobium, iron, tungsten) exhibit brittle features at low impact temperatures, and therefore, there is no deposition under such conditions. Figure 8d shows the particle size effect on the impact velocities and critical velocities. For most materials, there is an optimal size-range (region II, also named deposition window) where particle impact velocity is obviously higher than the critical velocity. In region I, no deposition or effective bonding would occur due to insufficient particle impact velocity, while in region III, impact velocity is quite close to critical velocity, resulting in low deposition efficiency and high porosity levels (Ref 16). The developed deposition window facilitates the selection of an optimized particle size distribution to manufacture high-performance deposits.

#### Polymer Surfaces

**Fig. 9:**
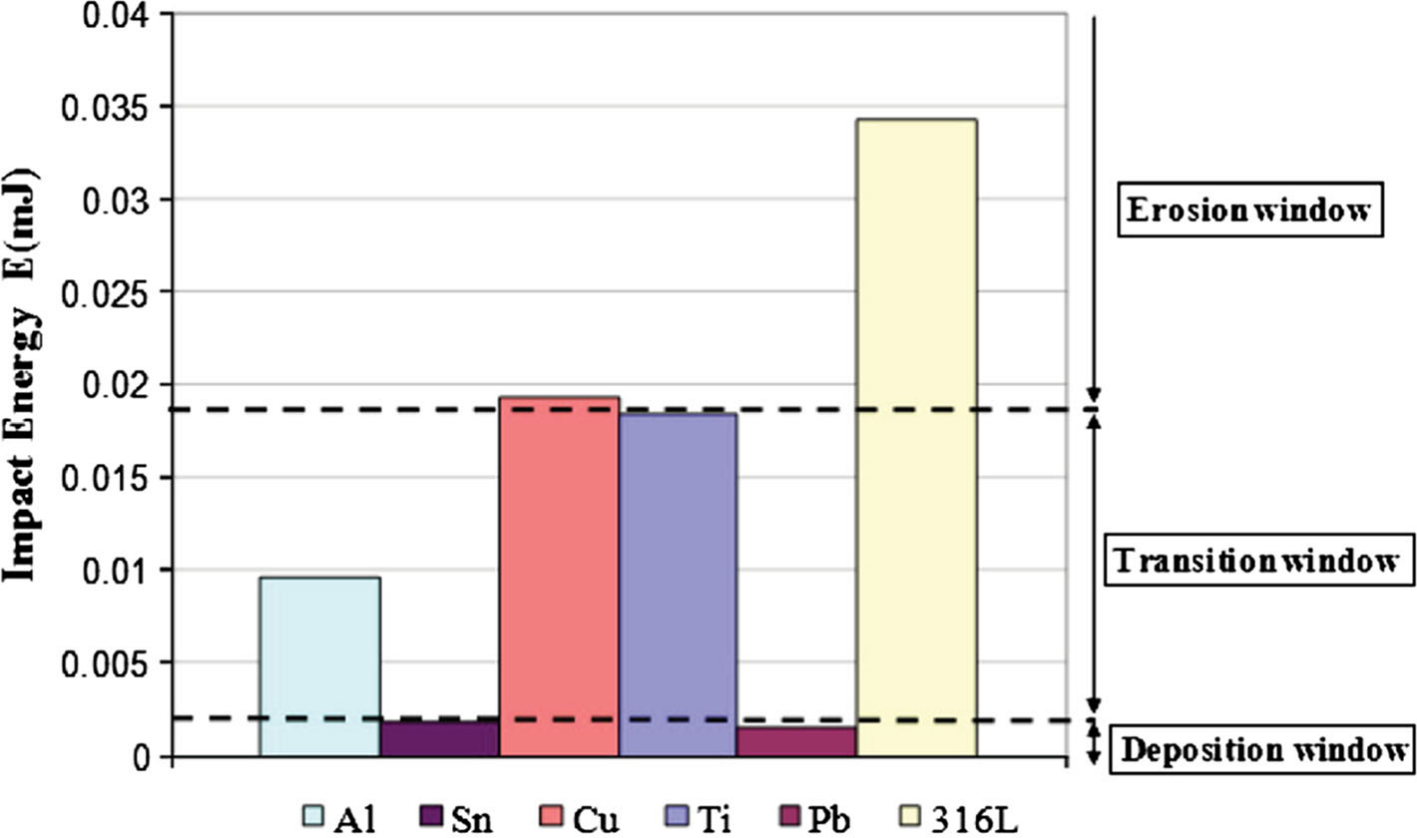
Impact energy of different material deposited onto polymer substrates (Ref 78)

The substrate characteristics have a great influence on the formation of the initial layers and to a certain extent on the subsequent deposits in CS. Deposition onto a relatively soft substrate, such as polymer or tin, will most likely lead to a damaged surface with craters. For example, attempt has been made to deposit Al particles onto acrylonitrile butadiene styrene (ABS), which has a hardness of 0.17 GPa. Although individual particles can be found on the ABS polymer surface, the main feature of the surface is the bombardment rather than deposited layers (Ref 76). However, the deposition of Al onto a carbon fiber reinforced polyaryl-ether-ether-ketone (PEEK) has been demonstrated (Ref 77) possible. Lupoi et al. (Ref 78) also investigated the feasibility of cold spraying metallic deposits (e.g., copper, aluminum, and tin) onto polycarbonate and ABS. It is found that Cu particles are able to embed into the plastic materials to form a first layer deposit under low gas pressure, while the subsequent metal-to-metal layers fail to form due to insufficient particle kinetic energy. When using higher processing parameters, the substrate surface experienced severe erosion and subsequent metallic layers cannot form. For tin, the deposition onto various polymer substrates was achieved, and coatings have been formed (Ref 78). This could be attributed to the low critical velocity and material density of tin. As shown in Fig. 9, the calculated impact energy of a single Sn particle is much lower than for Al and Cu under same processing parameters, and the velocity of Sn particle for deposition is reported to be as low as 200 m/s (Ref 16, 75, 78). Although the direct deposition of Cu particles onto polymer surfaces is difficult due to its high impact energy (see Fig. 9), CS coatings can be deposited onto polymer surface by using mixed Cu powder and PEEK powder as feedstock, and the coating exhibits a comparable electrical conductivity to pure Cu (Ref 79).

#### Pulsed Cold Spray

**Fig. 10:**
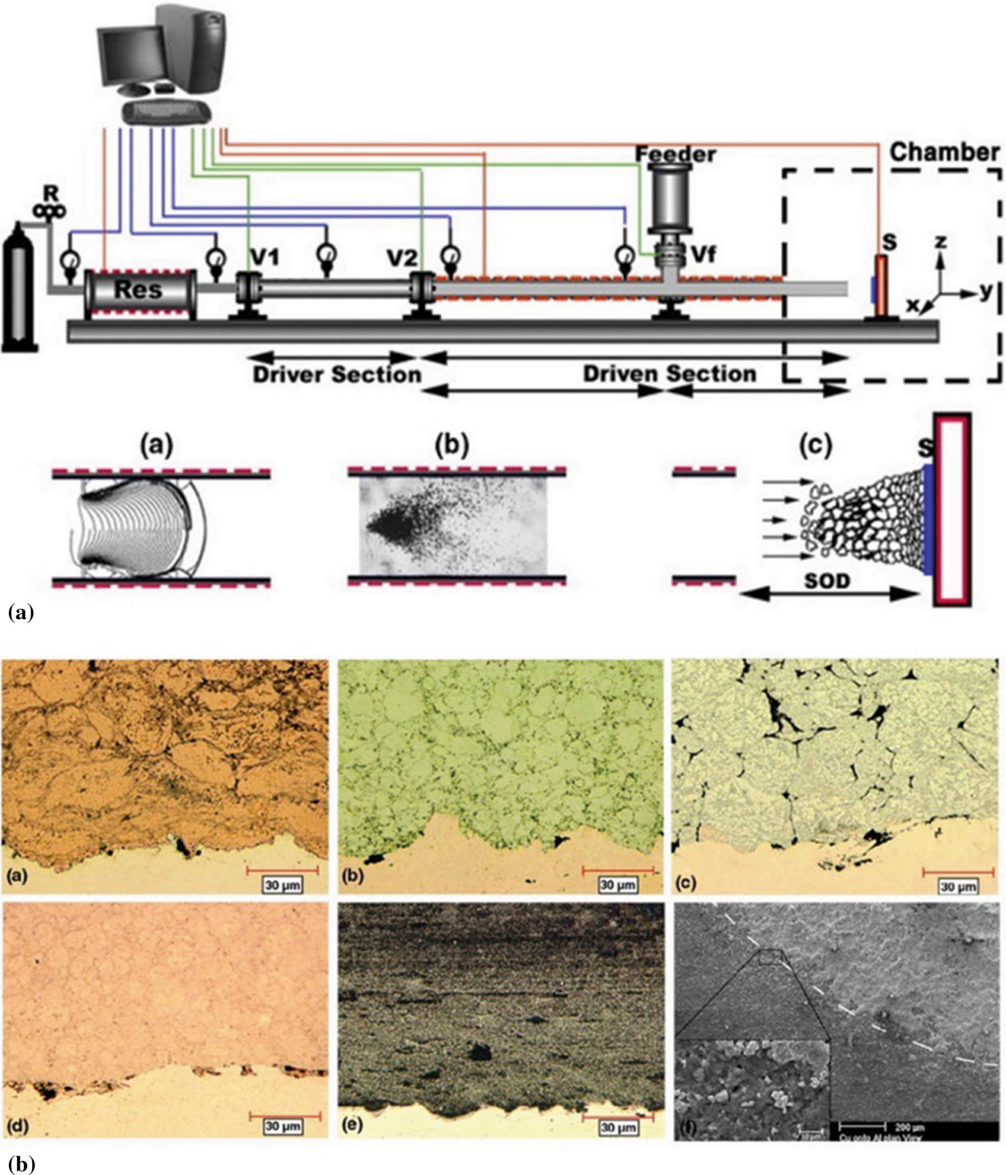
(a) Pulsed-gas dynamic spraying system and its working principal; (b) Microstructure of typical P-GDS coatings (Cu, Zn, Al, Al-12Si, and nanocrystalline WC-15Co) deposited onto Al substrate (Ref 54)

In CS, powder preheating is a common strategy to promote particle bonding as the critical velocity decreases with the increase of particle impact temperature. When a heated processing gas flows through a de-Laval nozzle, the gas temperature decreases in the diverging section. Therefore, the effect of preheating particles through preheating the working gas is not that prominent as expected. Based on CS, a new technology named pulsed-gas dynamic spraying (P-GDS) was developed in 2006 (Ref 54). Figure 10a shows the P-GDS system and its working principle. Compressed gas tank is connected to a regulator which controls the pressure in the shock wave generator between each pulse. The gas is heated in a heater, and a thermocouple is set at the exit to monitor and control the gas temperature. V1 and V2 are two high-frequency globe valves. These two valves and the confined space between them constitute a shock wave generator. Asynchronous opening and closing of these two valves enable the gas to produce pulse vibration of a certain frequency, and a shock wave is generated, as shown in inset (a). The feedstock powder is heated to a certain temperature prior to injection and accelerated by the moving shockwave without being cooled down as the supersonic flow created by the shockwave remains at high temperature as opposed to the flow in a converging-diverging nozzle. The accelerated particles impact and bond to the substrate to form a deposit (see inset (c)). Until now, a variety of materials (e.g., Cu, Zn, Al, and Al-based composite, WC-based cermet, Al-12Si, and its composite) have been successfully deposited by using the process (Ref 54, 80–84). Figure 10b shows the microstructure of typical P-GDS coatings (Cu, Zn, Al, Al-12Si, and nanocrystalline WC-15Co, respectively) deposited onto Al substrate. The result shows that the particles are deformed upon impact with the substrate, which is similar to CS. The Cu, Zn, Al-12Si, and WC-15Co deposits exhibit high density. The measured average Cu particle velocity (250 m/s) for successful deposition is below the reported critical velocity in CS (over 500 m/s) (Ref 54). This indicates the increased particle impact temperature can lead to a decreased critical velocity. P-GDS technique shows the capacity to manufacture high-performance deposits.

#### Laser-Assisted Cold Spray

**Fig. 11:**
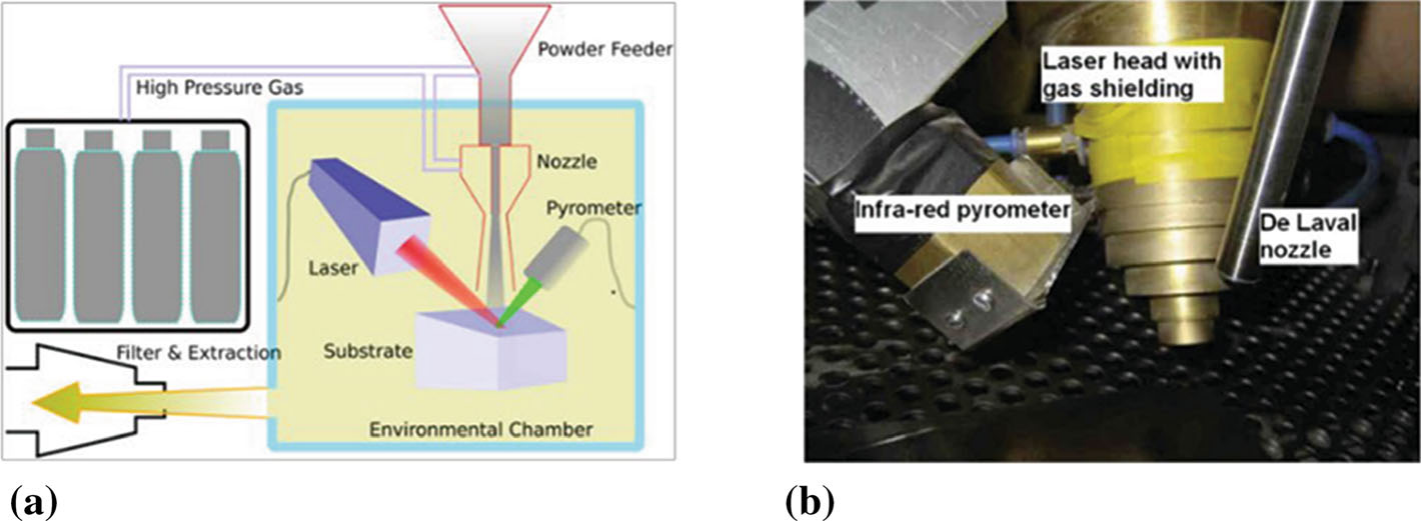
Laser-assisted cold spray system (Ref 85)

Laser-assisted cold spray (LACS), also named supersonic laser deposition (SLD), is a material deposition technique which combines the advantages of CS and laser irradiation (Ref 85). Figure 11 shows the LACS system. The deposition region is illuminated by the laser spot in order to soften the substrate material or previously deposited layers and incoming particles to a temperature below their melting temperature (Ref 85). The heat input from laser irradiation could soften the incoming particles, and therefore, these particles are able to deposit at lower critical velocities and experience more significant plastic deformation due to thermal softening effect.

LACS has been successfully applied in the deposition of some difficult-to-deform metallic materials, such as Ti (Ref 85, 86), Ti alloy (Ref 87), W (Ref 88), Ni60 (Ref 89), Stellite-6 (Ref 90–94), and their composites (Ref 95–99). The deposition of such materials by conventional CS usually leads to a high porosity, weak bonding, and poor mechanical properties of deposits. In addition, the feedstock powders are usually preheated before spraying, and helium is usually required as processing gas in order to achieve better particle acceleration and enhance particle plastic deformation, which significantly increase the manufacturing costs. With the help of laser irradiation, the particles are thermally softened and experience more prominent plastic deformation which enhances the atomic diffusion between interparticle interfaces and thus increases the interparticle metallurgical bonding (Ref 99). For example, the porosity of the LACS Ti deposits ranged from 0.3~0.6% which is much lower than that of conventional CS Ti deposit (2~4%). One of the concerns about laser heating is the oxidation of feedstock powder. However, the oxygen content of the LACS deposit was measured to be 0.6 wt.% which is close to CS Ti deposit. As for the mechanical properties of LACS deposits, available studies have suggested that the tensile strength of LACS deposits (e.g., tungsten (Ref 88) and stainless steel 316L (Ref 100) is close to their counterparts manufactured via conventional processes.

#### “Micro” Cold Spray

**Fig. 12:**
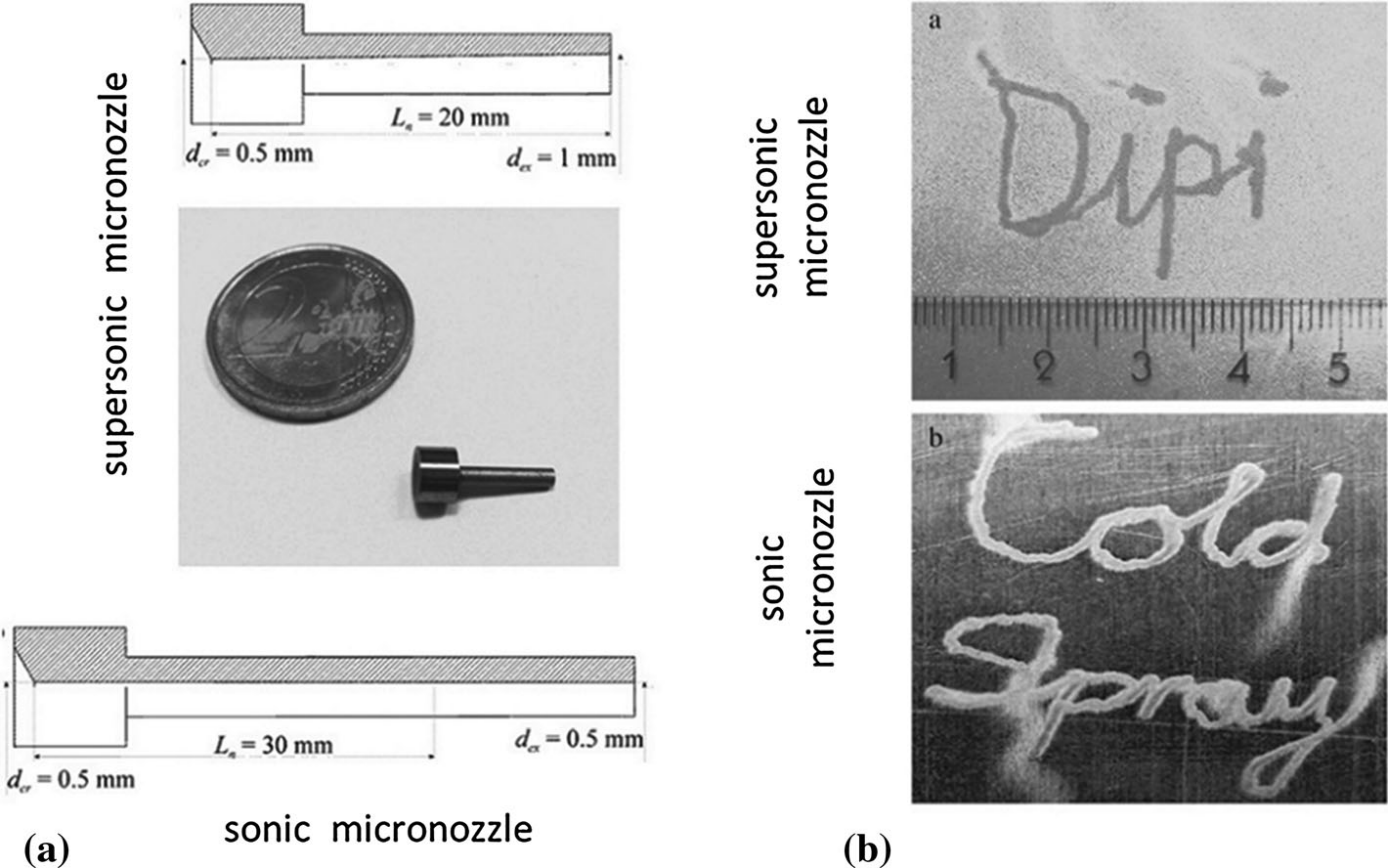
(a) Schematic view of supersonic and sonic micro-nozzles; (b) Cu deposits on Al substrate sprayed by supersonic and sonic micro-nozzles (Ref 101, 102)

The nozzle is the core part of a CS system, where feedstock particles and propulsion gas are mixed and accelerated. CS nozzles can have different shapes and configurations, such as cone shape, bell shape, plate shape, and shock shape (Ref 11, 92–96). In general, the nozzle exit diameter ranges from 5 to 10 mm, which limits the spatial resolution of CS (Ref 101). To overcome this limitation, micro-nozzles with low exit section areas (< 1 mm^2^) were developed, as shown in Fig. 12a. It is notable that the gas flow rate through such micro-nozzles is 10 times lower than conventional CS nozzle, with such nozzles also being characterized by a limited length. Under these conditions, using helium as processing gas is often the only possible solution to reach particle deposition speed. Figure 12b shows Cu deposits onto Al substrate using micro-nozzles. The deposits have a 1-mm-width and a thickness of only 20~30 μm, which could be attributed to the insufficient particle impact velocity. Moreover, it is reported (Ref 102) that the porosity of CS Al deposit by micro-nozzle is near 4.5-5%, which is much higher than that of conventional cold spray Al deposits. The high porosity leads to low adhesion strength and poor mechanical properties of deposits. More efforts should be made on the process parameters and particle size distribution optimization and development of novel gas heater for low gas flow rate in the near future. If the current problem is well-explored this novel technology will be expected to be applied with some success in the direct printing technologies, such as electrical circuits manufacturing.

#### The “tamping” Effect

**Fig. 13:**
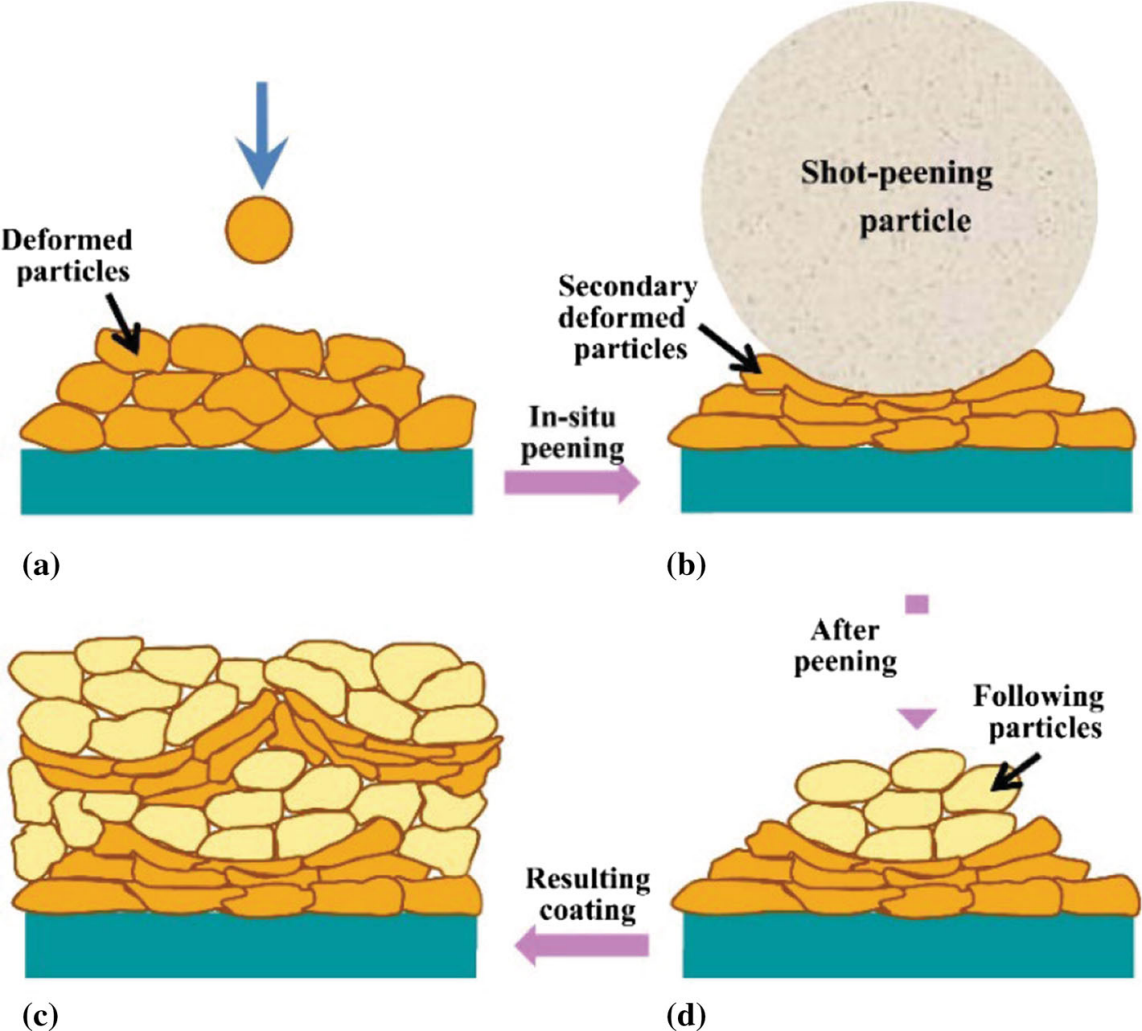
The illustration of in-situ shot peening-assisted cold sprayed deposition mechanism (Ref 104)

In CS, the previously deposited layer undergoes the impact of subsequent incoming particles, which further promotes the plastic deformation of this initial layer and thereby densifies the deposit. This effect is known as tamping effect, which is similar to the cold working process of shot peening. To intensify particle deformation and enhance this tamping effect, some researchers proposed to blend large size shot peening particles (typically 100~300 μm) with high hardness, such as stainless-steel powder, into feedstock. Figure 13 shows the illustration of in-situ shot peening (also known as in-situ tamping, in-situ hammering, and in-situ micro forging) assisted CS deposition mechanism. Large-sized shot peening particles are carried by the high-velocity gas-flow and compact the deposited layers to enhance plastic strain and reduce voids between interparticle boundaries. The impact velocity of shot peening particles fails to reach their critical velocity for deposition due to their large sizes, and therefore, they rebound from the substrate or previously deposited layer after impacting, which is theoretically capable to avoid contamination of shot peening particles inclusions (Ref 103).

**Fig. 14:**
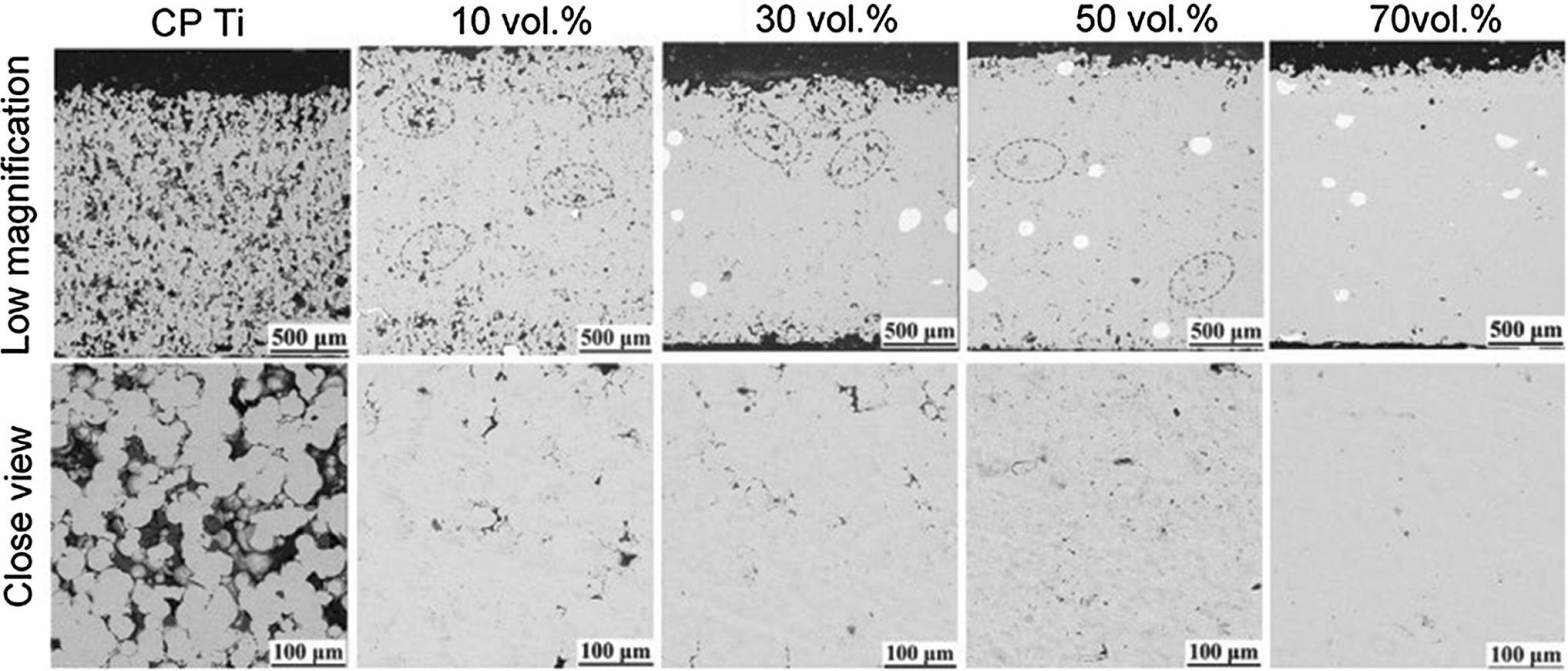
Cross-sectional microstructures of the titanium coatings deposited with pure titanium powder and powder mixtures with different proportions of shot peening particles (Ref 105)

Figure 14 shows the cross-sectional microstructures of the Ti64 coatings deposited with Ti64 powder and powder mixtures with different proportions of shot peening particles, which were fabricated using nitrogen as propulsive gas. The density of the deposit significantly increases with the increasing proportion of shot peening particles, and the porosity can decrease to less than 1% which is comparable to that of conventional CS deposit using helium (Ref 105). In-situ shot peening assisted CS favors well-bond interparticle interface and less voids, even with nitrogen as processing gas. The introduction of foreign powder may partially embed in the deposit and lead to undesirable stress concentrations and localized chemical heterogeneities, especially when a high proportion of shot peening particles are blended for achieving a nearly full-dense deposit. In addition, the additional powder mixing procedure also increases workload and manufacturing cost (Ref 106).

#### Intermetallics in Cold Spray do Exist

**Fig. 15:**
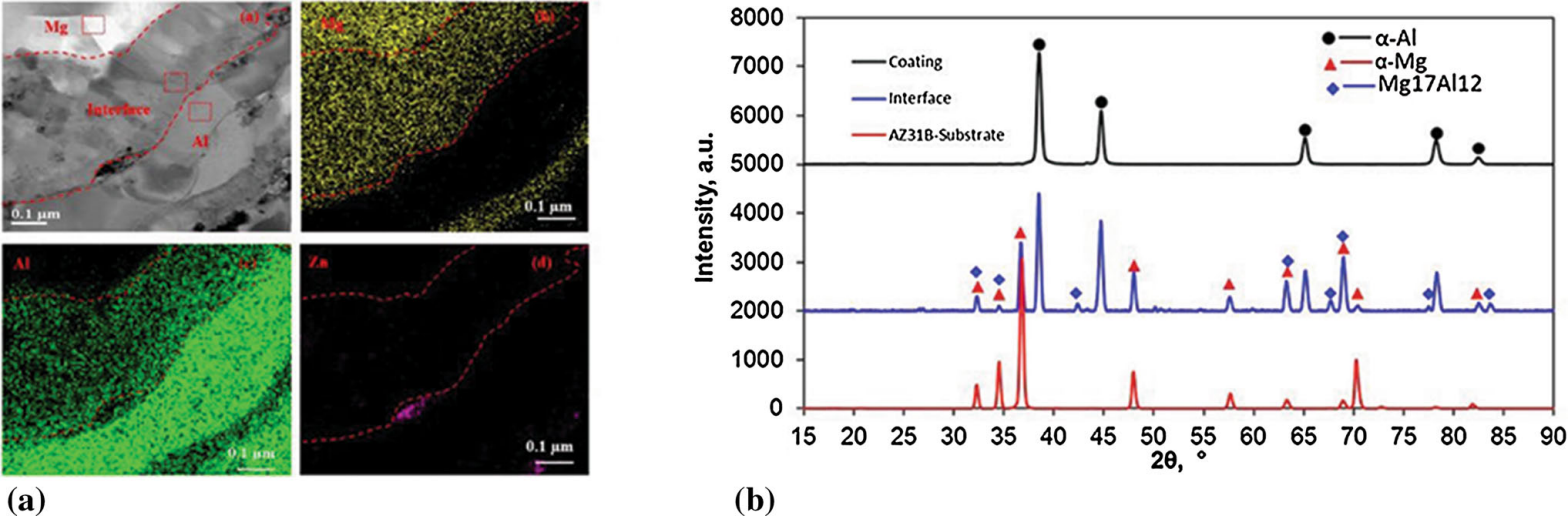
(a) Bright field-transmission electron microscope image with EDX mapping and (b) XRD patterns at the interface between AZ31B Mg alloy substrate and AA7075 alloy deposit (Ref 109)

The bonding mechanisms of metallic particles in cold spray process have been always an important research topic. Localized metallurgical bonding is considered to be the result of the contact interface reaching the melting point of the materials, which mainly occurs in the process of cold spraying materials with low melting temperature (e.g., Zn and Al). The fusion phenomenon during cold spraying of various materials including Al-12Si, Ti, Ti-6Al-4V, Ni, and NiCoCrAlTaY has been reported for certain impact conditions. Low melting point of spraying materials, relatively high gas temperature and chemical reaction with the atmosphere are considered as the main factors resulting in the impact fusion (Ref 107). Another evidence of impact induced localized melting is the intermetallic at interparticle interface or particle/substrate interface. Ni_3_Al was identified by XRD at the interface between Al deposit and Ni substrate (Ref 108). Moreover, the Mg_17_Al_12_ phase with α-Al and α-Mg phases have also been recognized after cold spraying AA7075 alloy onto AZ31B Mg alloy substrate, as evidenced by the EDX mapping and XRD pattern at the interface in Fig. 15 (Ref 109). In brief, intermetallics are definitely there in cold spray process.

#### High-Speed Videos of Particle Impacts

**Fig. 16:**
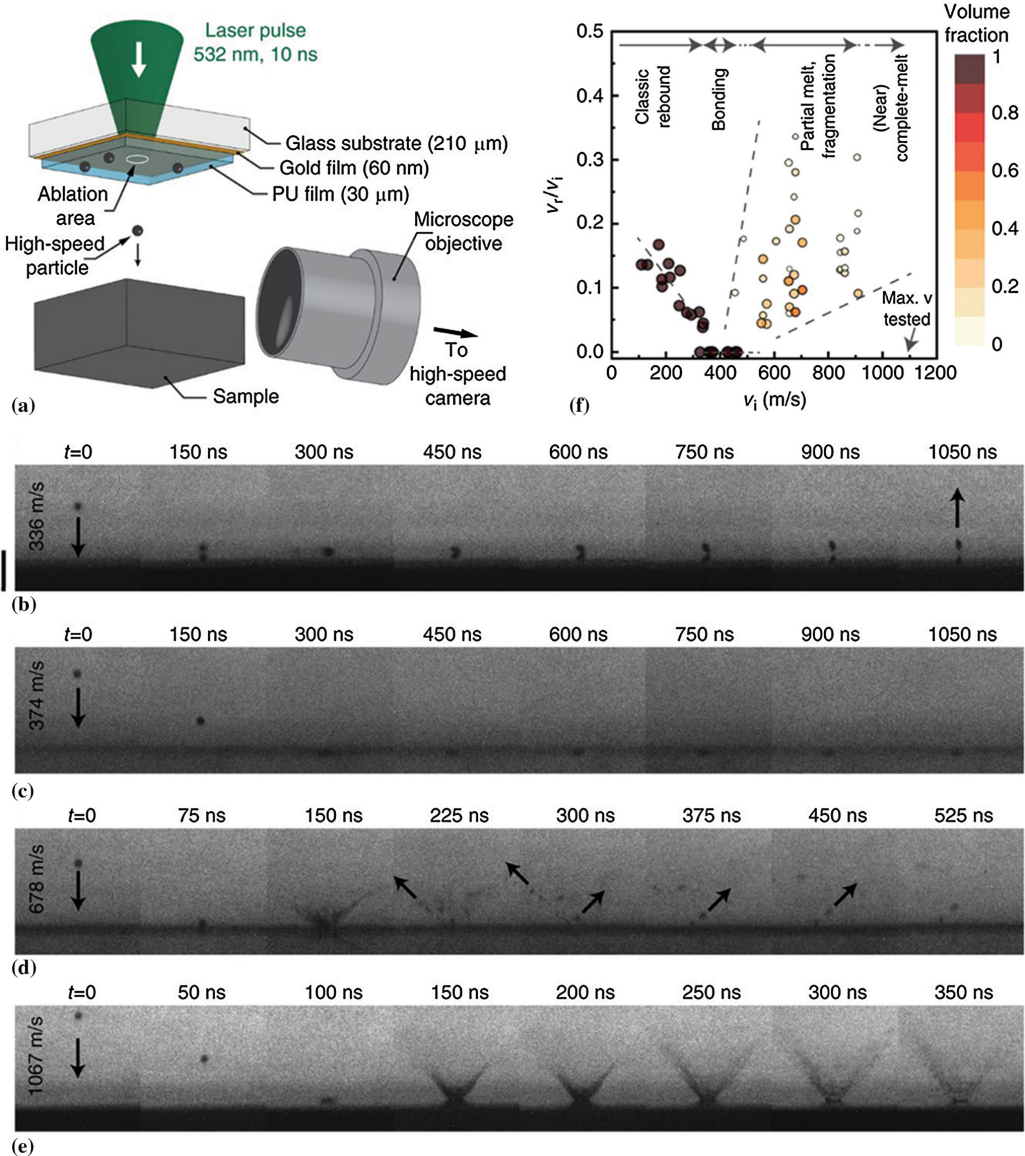
(a) Experimental setup of the microparticle impact test and real-time high-speed imaging system; (b)-(e) Multi-frame sequences with 5 ns exposure times showing the process of tin particles approaching and impacting onto tin substrate at increased velocity, spanning from the rebound regime to the bonding and the erosion regimes; (f) Coefficient of restitution, v_r_/v_i_, of the rebounding tin particles and fragments. (Ref 110)

The observation of particles impacting onto target surfaces has been challenging in CS due to the short timescales and particle size involved. Although the impact behavior of CS particles can be studied through numerical simulation, there is still shortage of experimental validation limiting our understanding on the deposition mechanism. Recently, a laser-induced particle impact test system was adapted to capture CS particles impacting onto a surface (Ref 110, 111). As shown in Fig. 16a, a laser excitation pulse is focused onto a pad assembly from which single particles are launched toward a target sample. The particle is accelerated to a “cold spray” velocity prior to impact with the substrate. The impact process is observed in real-time with a high-frame-rate camera and a synchronized quasi-cw laser imaging pulse for illumination. Tin particles impacting onto tin substrate are shown (see Fig. 16b-e). It exhibits the impact behavior of micro-particles rebounding from the target surface at low impact velocity (Fig. 16b), bonding with the target surface at a velocity beyond critical velocity (Fig. 16c), and eroding the target above erosion velocities (Fig. 16d-e). By calculating the coefficient of restitution (v_r_/v_i_), the bonding, rebound, and erosion regimes can be established, as shown in Fig. 16f. The developed system not only enables to find the critical velocity for a given particle, but also provides an enhanced understanding of impact behavior and bonding mechanism of CS particles.

#### Cold Spray Coatings can have Oxides

**Fig. 17:**
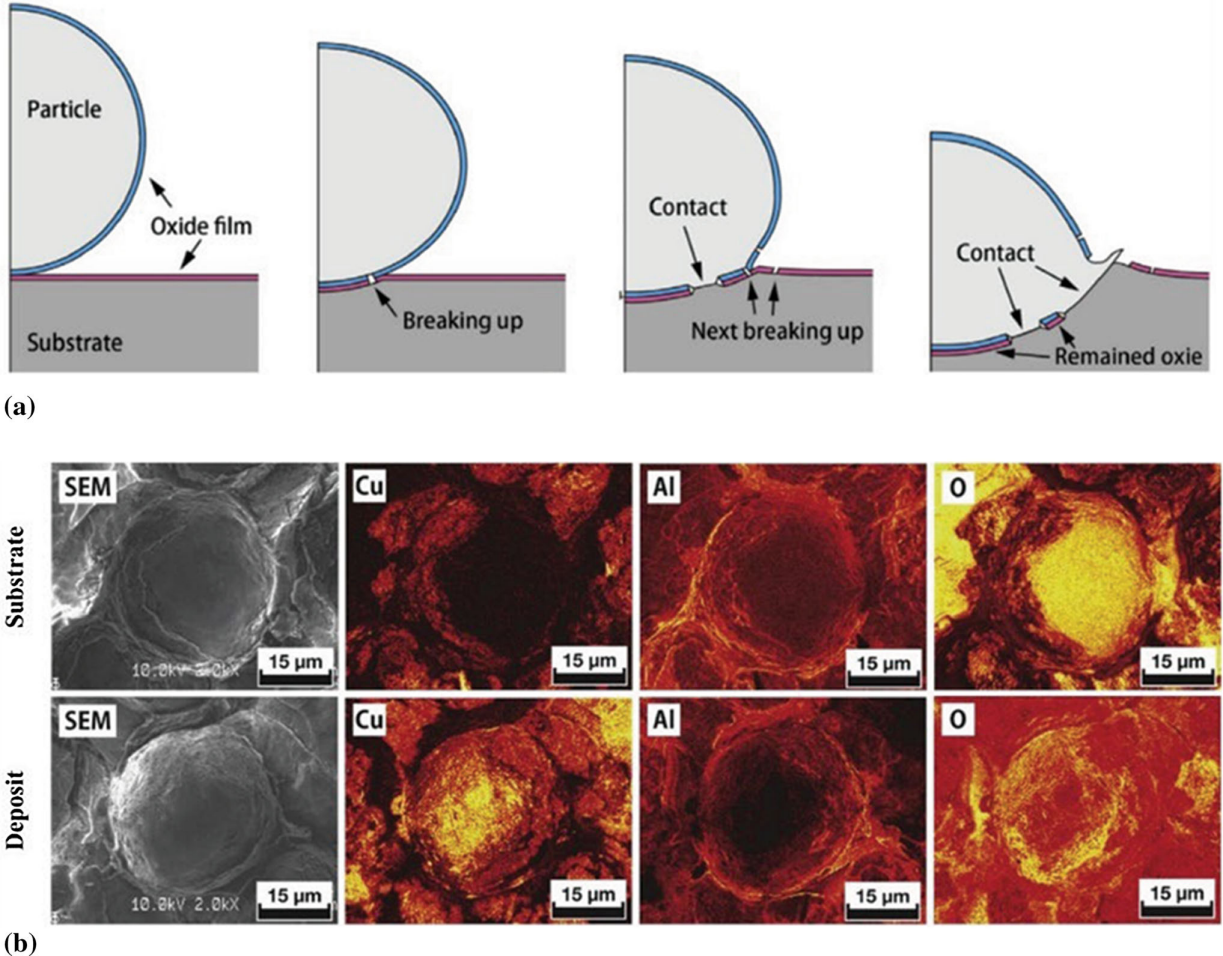
(a) Deformation of particle upon impact and break-up of oxide films (Ref 112, 113); (b) SEM images and AES mapping of fracture interface between substrate craters and deposition particles (Ref 112)

The deposition mechanism of spraying particles is a matter of the utmost importance to understand the CS deposits’ build up. One of the various proposed deposition mechanisms is the break-up of native oxide films, which must be removed/cleaned upon particle impact to allow proper contact between newly exposed metallic surfaces, as shown in Fig. 17a. When incoming particles impact onto the substrate, there is no shear plastic deformation at the “South Pole”. Therefore, the oxide films at that position remain intact, as confirmed by the high oxygen content at the center of the crater, shown in Fig. 17b. While the amount of oxygen at the interface away from this South Pole is quite low, which indicates the break-up and removal of the oxide film due to the large shear plastic deformation. In practice, however, some oxide films at fracture surfaces remain, and their presence is inhomogeneous due to different particle impact behaviors, such as impacting angle, surface morphology of particles, subsequent particles impacting onto the craters formed by previous rebound particles to name a few. The existence of these oxide films will be detrimental to the bonding between particle/substrate or interparticle, leading to decreased overall adhesion strength (Ref 112). Moreover, the oxides in CS deposit also result in heterogeneous microstructure, which brings low ductility even for high density coatings. By further enhancing the plastic deformation of deposited particles, such as with in-situ shot peening assisted cold spray or by increasing the particle impact temperature, the oxide films at the interparticle interfaces can be broken up, and better interparticle bonding can be achieved.

#### Bulk Additive Manufacturing

**Fig. 18:**
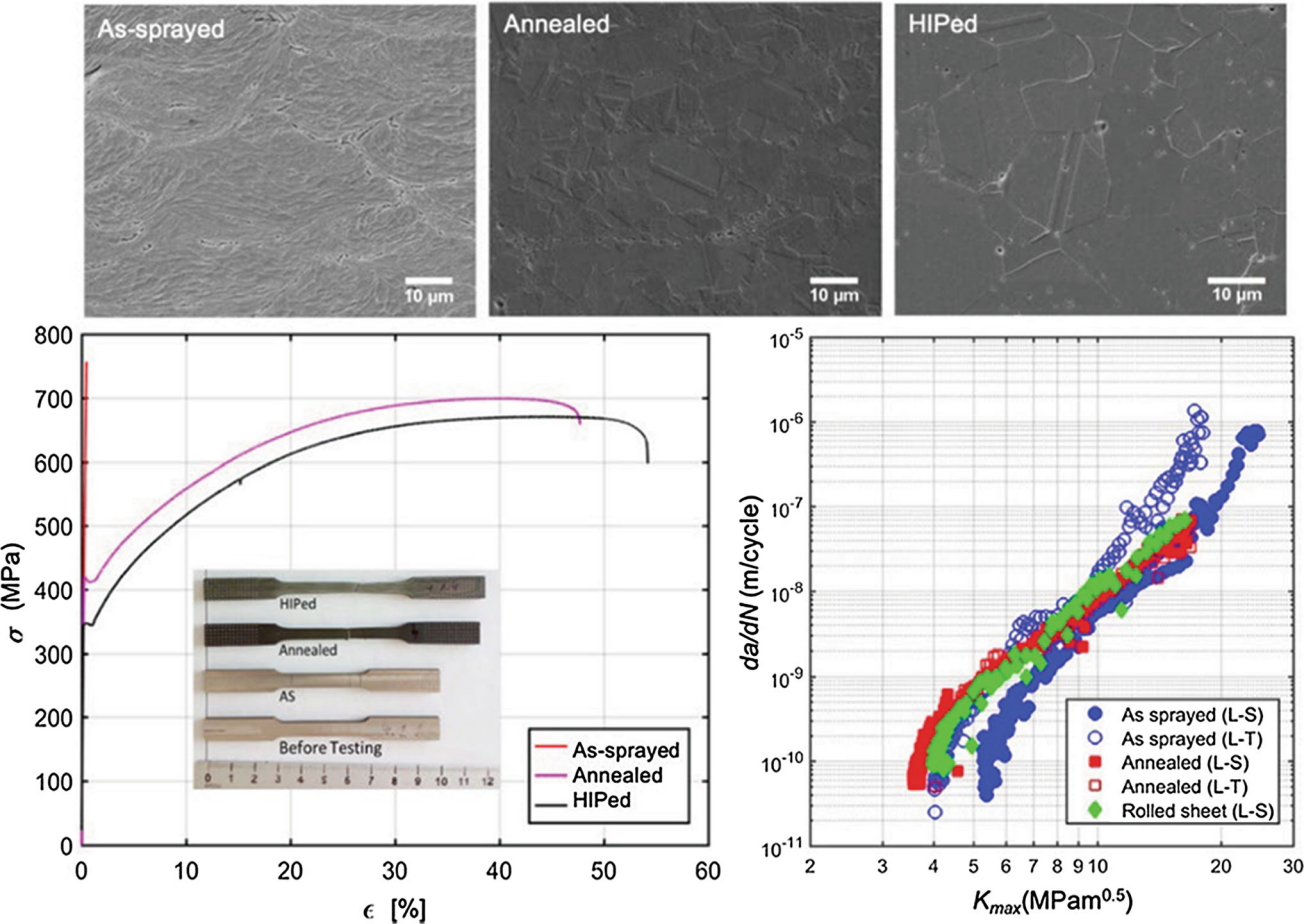
Cross-sectional microstructure, tensile properties, and fatigue crack growth rate of the cold sprayed 316L deposits before and after heat treatments (Ref 114)

While developed as a coating technology, CS strides into additive manufacturing to fabricate free-standing parts and repair damaged components. Compared with prevailing fusion-based additive manufacturing techniques (e.g., selective laser melting (SLM), laser metal deposition (LMD), laser beam melting (LBM)), CSAM retains all the advantages of CS, and it is able to fabricate large size parts. In addition, CSAM is particularly suitable for the manufacturing of high-reflectivity metals such as copper and aluminum (Ref 5). However, CSAM has some drawbacks/challenges in manufacturing parts with a more complex geometry, and post-machining is generally required like any other AM processes. Moreover, due to the existence of inherent defects (e.g., porosity and unbonded interparticle boundary), as-sprayed deposits normally have degraded mechanical properties in their as-fabricated state, such as lower ductility. In order to trade off strength for improved ductility, post-heat treatment, as an economical and efficient strategy, is commonly applied for as-sprayed deposits to promote interparticle metallurgical bonding and recrystallization. Figure 18 shows the microstructure, tensile properties, and fatigue crack growth rate of the CS 316L deposits before and after heat treatments. Original splat boundaries are visible for the as-sprayed deposits while the samples after heat treatment show more homogeneous microstructures due to atomic diffusion, associated recrystallization, and grain growth (Ref 114). Moreover, concerning the tensile properties, although as-sprayed deposits show no elongation, the ductility can be completely recovered after post heat treatment.

### Process Hype Cycle and Direction

**Fig. 19:**
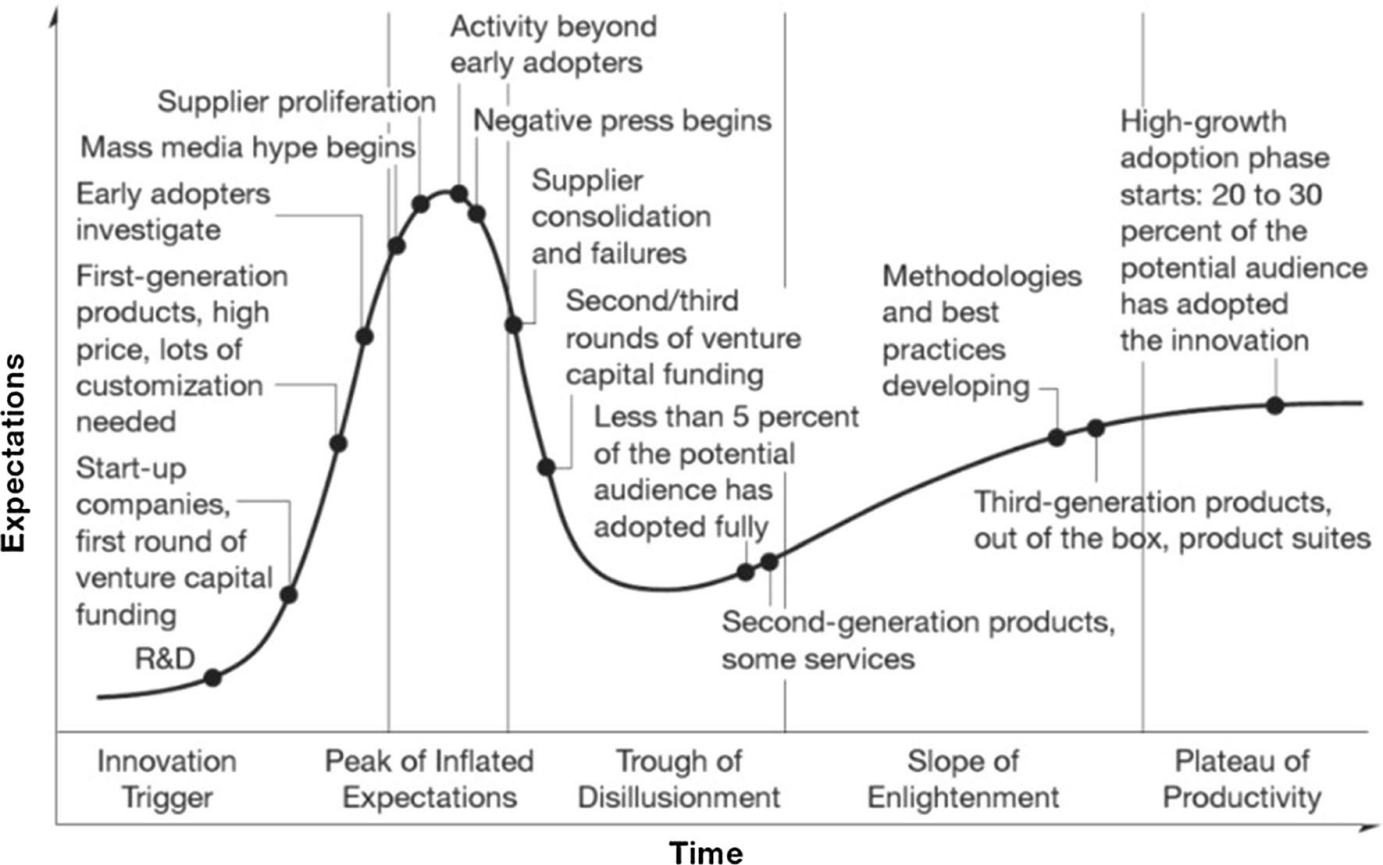
Stages and key indicators of hype cycle curve (Ref 115)

Cold spray conception and development has followed a path of more than 30 years. The expectations and the engagement of scientific community on the particular technique can be simulated by a “hype cycle model” as it has been developed by Gartner Inc. (Ref 115) to describe the evolution of technological innovations from conception to maturity. The hype cycle curve theoretically (Ref 116) consists of the following stages as presented in Fig. 19: the innovation trigger, the peak of inflated expectations, the trough of disillusionment, the slope of enlightenment, and the plateau of productivity.

The aim of the current section is to describe the trend of hype cycle expectations as research evolved until nowadays rather to track the development of the technique itself within the years.

#### Toward the Peak of Inflated Expectations

The accidental observation of the consolidation of aluminum particles in high-speed flows was the spark that inspired scientists to start developing the contemporary CS technique in early 80s (Ref 18). As the potential of a novel coating technology was appealing, researchers conducted their investigations focusing primarily on the process development through equipment improvement for enhanced reproducibility and reliability and secondarily on the understanding of the CS principles. The experimentation with air, argon and helium as propellant gases appeared promising in terms of processing flexibility and applications perspective (Ref 20). The advances in cold spray-related research led to the establishment of a company under the name Obninsk Center for Powder Spray (OCPS) to merchandise DYMET® CS equipment and accessories (Ref 117). As a result, CS started to expand toward the formation of coatings with acceptable structural integrity using several feedstock powders including iron, nickel, and titanium (Ref 22). Furthermore, early investigations demonstrated that the porosity level in CS coatings could remain in substantially low levels for certain materials (Ref 118, 119) and spraying conditions. The commercialization contributed to the adoption of the CS technique at industrial level. Moreover, the publishing of cold spray-related researches followed an exponential growth after 1994. This can be attributed to two main factors: firstly, the emigration of scientists outside Russia following the dissolution of the Soviet Union and secondly the rapid expansion of digitalization. Large enterprises and institutions in US and Canada started to show growing interest (Ref 120) around CS developments that resulted in significant flow of capitals aimed to enhance the applicability of the method. Studies performed in the late 90s showed that CS deposition efficiency could reach 95% in certain cases (Ref 121), a finding that boosted the expectation for the economic sustainability of the technique and its adoption in the mass production of coatings at industrial level. Experimental and numerical investigation demonstrated that CS copper coatings (Ref 122) are characterized by the absence of fusion of powder particles that confirmed earlier assumptions (Ref 38). As a result, CS started to attain an attractive melting-free character contrary to traditional thermal spray techniques. The elimination of heat-affected zone was considered to be a unique feature of paramount importance from a metallurgical aspect, as it is usually the area where undesirable microstructural transformation may occur. Moreover, processing of cermets (Ref 123, 124), the production of commercial portable CS device (Ref 125), and improvement of equipment (Ref 11) were milestones of the process development until the early 2000s. Apart from thermal or electronic device applications, CS started to be used for the formation of tribological coatings, with high resistance against corrosion (Ref 126) and wear (Ref 127) and significant applications in automotive, aerospace, and chemical sectors. After 2002, CS variants with additional technological principles started to appear aiming at porosity elimination and deposition efficiency enhancement. The most notable of them were pulsed-gas dynamic spraying (PGDS) (Ref 54), kinetic metallization (Ref 48), and LAMS (Laser-Assisted Material Spray) (Ref 51). Despite early CS researches focused on metals, later studies demonstrated that metallization of polymers and composite materials (Ref 128, 129) was feasible due to the “cold” character of the technique. Researchers’ comprehension (Ref 130, 131) that CS was more than a simple coating technique, and that it can follow a layer-by-layer strategy similar to AM methods, further boosted the interest of academics and markets at the onset of Industry 4.0 (Ref 132). The peak of inflated expectations of CS can be estimated to have occurred around 2010 in the sense that until then the publications showed a constant trend in presenting the advantages of the technology. It is characteristic that in the proceedings of ITSC 2012 Giraud et al. (Ref 128) highlighted the wide interest in CS mentioning that “there is justified craze for cold spray”.

#### Trough of Disillusionment

As the CS technology started to develop globally, it was not long before researchers began to comprehend the intrinsic limitations of the technique and therefore to investigate innovative methods to overcome them. Some early indications of CS drawbacks can be found even in publications earlier than 2010 (Ref 133). A major drawback was stated to be the consumption of propellant gas that is commonly higher (1000-3300 Nlm/min) compared to other thermal spray techniques such as plasma spray (40-150 Nlm) and HVOF (400-1100 Nlm) (Ref 134). Furthermore, when experimentation on hard materials started (Ref 135) helium use was highly desirable due to the resulting high flow speed that is about 2.5 times faster than nitrogen (Ref 136). It is a characteristic that several publications of that time (Ref 137, 138) (2004-2010) mentioned helium as the primary gas for CS. However, it was not long before scientists comprehended that the use of helium increased significantly the process cost (Ref 139) considering that it is approximately ten times more expensive than nitrogen (Ref 140) and particularly uneconomic when spraying large surfaces or fabricating parts (as AM technique).

The efficiency of the method to process certain materials was hindered due to nozzle clogging mechanism that prevented the operation of CS at high gas preheating temperatures. Several researchers (Ref 141–144) investigated potential solutions focusing on the application of an efficient cooling system that prevents the overheating of the nozzle walls. Despite this being a clear controversy as a level of energy is added to raise the gas temperature, and now, a portion of it is removed to cool the nozzle, it has been proven as an efficient and practical way to avoid nozzle clogging (particles deposition in the inside channel) and is nowadays adopted in several CS commercial systems. An additional consideration concerning CS is the bonding strength between the coating and substrate (Ref 145), which is often weaker, compared to thermal spraying techniques that involve fusion. When used for coatings fabrication, CS coatings were mainly examined in terms of electric and tribological properties. However, after the adoption of CS as an AM technique of metallic parts, the obtained bulk properties started to be under the microscope. In as-sprayed condition, strength and ductility were found to be degraded (Ref 146) while in certain cases a thermal or mechanical post-processing aiming at their enhancement was suggested. Another drawback that gradually attracted researchers’ attention was the high roughness (Ref 147-149) of the CS coatings that needed minimization for practical or esthetic reasons. Furthermore, despite thermal stresses remaining at low levels in CS, the intense plastic deformation was found (Ref 150) to result in high residual stresses (Ref 151) that could lead to delamination or crack propagation during or after the CS process. Finally, an intrinsic drawback is the low geometrical accuracy of the final parts (Ref 130) that is mainly related to the size turbulences of the gas stream and the large size of the nozzle.

It can be concluded that the trough of disillusionment of CS hype cycle despite carrying a certain amount of disappointment for the scientific community, was a meaningful period where the drawbacks of CS emerged along with alternative and innovative solutions. Table. 1 indicatively summarizes these drawbacks, some proposed actions for their elimination, and further complications that they have created.

**Table 1:** Investigation of cold spray drawbacks and potential solutions in the course of trough of disillutionment

Cold spray drawbacks	Potential solutions	Additional complications	Indicative references
High gas consumption/helium cost	Gas recycling	Recycling efficiency, cost increase	(Ref 40, 47)
Low deposition efficiency	Powder recycling, helium use, gas preheating, variables optimization	Cost increase	(Ref 152, 153)
Weak bonding between coating-substrate	Helium use, gas preheating, post-processing, surface roughening	Cost increase	(Ref 154-156)
Nozzle clogging	Cooling system, optimization of powder injector, optimization of nozzle design, powder optimization	Cost increase	(Ref 157, 158)
Degraded mechanical properties	Post-processing	Time consuming, cost increase	(Ref 5, 7)
High roughness	Optimization, machining	Time consuming	(Ref 147, 148)
Low Geometrical accuracy	Optimization strategies, nozzle angle control	Time consuming	(Ref 151, 159)
High Residual stresses	Post-processing	Time consuming, cost increase	(Ref 160, 161)

#### Slope of Enlightenment

It is obvious that the CS journey over the years has many uncertainties and is difficult to make definite conclusions regarding its current global trend, especially in a post-Covid world. However, the authors claim that its present status lies inside the slope of enlightenment. The increased interest is not related only to the improvement of the relative techniques that alleviate its drawbacks, but also to several additional positive factors that currently contribute to its rebirth. Firstly, CS has found applications in lucrative sectors such as oil & gas (Ref 162), biomedical (Ref 163, 164), and nuclear waste management (Ref 165, 166) industries. Secondly, the repair of parts with CS showed an attractive potential to extend the lifespan of high value parts (Ref 5, 7). Thirdly, as the cost of CS equipment decreased, its market increased over the years in terms of vendors as well as clients (Ref 167, 168). The network of institutes, research centers, universities, and companies that supply CS hardware and related products (e.g., powders, gasses) expanded along with the knowledge exchange between them.

#### Plateau of Productivity

**Fig. 20:**
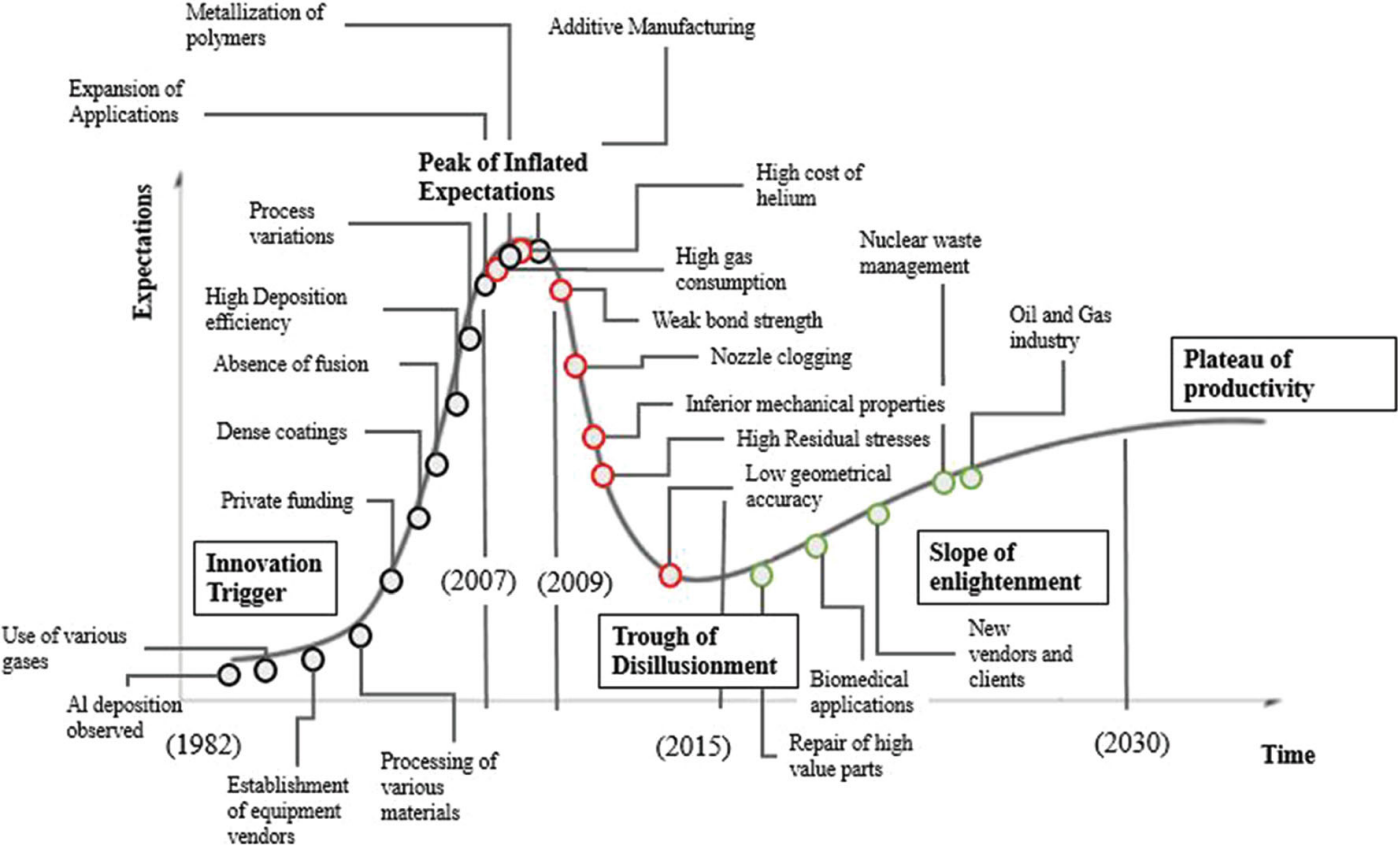
Indicative hype cycle of the cold spray technique

It is difficult to predict when or how CS will reach its plateau of productivity as new uncertainties arise and interdisciplinarity of research projects expands. Shifting of manufacturing toward a sustainable future dictates the growing need of CS use due to its green character. Moving from slope of enlightenment to plateau of productivity is expected to expand CS use in the aerospace sector in order to replace or complement fusion-based techniques such as plasma and HVOF. Quest for funding will mainly determine the growth around CS research and its development to an entirely industrially incorporated technique. The scientific findings originated mainly during the trough of disillusionment are expected to contribute toward the CS maturity and its establishment on a realistic basis as the lastborn child among the other members of the thermal spray family. In a greener world (that is in the short future), CS will likely find additional applications. The fact the process does not have a fusion character, its carbon footprint is reduced and as the other processes come under pressure due to high emissions, CS will find a new niche. A positive move in biomedical applications is expected, an area that tightly regulated. Literature evidence suggest potential breakthroughs (i.e., in the polymer metallization topic), but these will not turn into industrial relevant research until CS becomes certified. Other spray processes are, however, reaching their developmental limit, and as the future will need more process control and environmental compatibility, we do expect more interest in exploring process-specific certifications. The indicative hype cycle curve as adjusted for the birth and evolution of the cold spray technology is presented in Fig. 20.

## Cold Spray Application Pillars (Past, Present, and Future)

### Cold Spray of Copper and Pure Aluminum

Copper and aluminum have high thermal and electrical conductivities and useful corrosion resistance properties. Both materials are extensively used in heat sinks for microelectronics (Ref 169), metallization of polymer substrates for aerospace applications (Ref 170), corrosion resistance in harsh urban, industrial and marine environments (Ref 171, 172) and as matrix materials in composites (Ref 173). The popularity of both temperature sensitive materials in the CS field is associated to these industrial applications, their relative ease of deposition by CS, and most importantly to the absence of high temperature particle heating, which reduces oxidation, promotes retention of original material properties, and reduces detrimental residual stresses in the deposition. Additionally, the high reflectivity of both materials make them challenging for laser-based additive manufacturing (AM) processes, consequently CS stands as a potential alternative for AM of copper and aluminum components (Ref 174). The renewed interest of copper coatings for antimicrobial, antibacterial, and antiviral applications has also led to many CS studies (Ref 175, 176). Moreover, the common method used by many nations to store used nuclear fuel bundles is through storing them in copper cylinders machined from large ingot down to size. In Canada, the plan for the long-term and safe management of used nuclear fuel in underground repositories will be relying on copper-coated steel containers that incorporate a CS copper layer at the closure weld region of the used nuclear fuel containers (Ref 177). This is likely to be one of the most intensive applications of the CS technology for a few decades when the project officially starts its production phase.

In addition to the extensive use of both materials in numerous applications, copper and aluminum have also been significantly utilized for the fundamental study of high strain rate material deformation processes (Ref 178, 179), the development of numerical constitutive models (Ref 180, 181), and the analysis of defects evolution through molecular dynamics (MD) simulations (Ref 182). These materials have a face-centered-cubic (FCC) structure, which allows greater particle deformation and bonding at lower critical velocities than other crystalline structures. Additionally, due to their different stacking fault energies (SFE) and melting temperatures, the influence of dislocation densities on recrystallization processes during impact have been heavily investigated (Ref 178, 183). The ease of deposition along with the inherent material properties of both Cu and Al has led to the discovery and understanding of recovery and static and dynamic recrystallization during high-speed impacts (Ref 178).

Since both copper and aluminum’s properties are better established than other materials, many studies choose these metals to conduct fundamental studies and investigation on the underlying mechanisms involved in deformation processes (Ref 184) and dislocation activity at the atomic level during high-speed impacts (Ref 185). Thus, much of the established knowledge base and understanding of CS deposition mechanisms has originated from studies on copper and aluminum (Ref 59). The deposition process in CS relies on the kinetic energy of particles upon their impact onto the substrate. Hence, the deformation of both particle and the substrate play the major role in depositing the powders. The existed knowledge about the deformation behavior of copper and aluminum at high strain rates, enabled to explain and understand some mechanisms involved during the deposition process in CS.

When soft materials, such as aluminum, copper, and magnesium, are sprayed onto similar soft materials, the chance of creating both metallic and mechanical bonding is high. On the other hand, the chance of bonding decreases when these soft materials are impacted onto harder substrates, such as hardened steel. In this case, the soft particles cannot deform the target surface to generate mechanical anchoring, instead, the only mechanism is metallic bonding (Ref 184, 186). In the dissimilar hard/soft and soft/hard pairs, the sequence of particle impact influences the deformation and bonding of the particles (Ref 187). Therefore, in order to increase the particle adhesion on hard substrates, surface preparation processes, such as grit blasting or pulse-water-jet, are used frequently to create surface asperities that facilitate mechanical anchoring (Ref 186).

### Bioactive Materials and Surface Sanitation

Bacterial infections are a major threat to human health resulting in high mortality rates (Ref 188-191). Despite efforts and advanced development in antibiotic agents, healthcare infection problems are persistent and are associated to pathogens antibiotic resistance (Ref 190). In addition, the antibiotic effectiveness is reduced significantly once the bacteria form a biofilm (Ref 192). Therefore, it is important to develop alternative solutions to prevent bacteria attachment and biofilm formation. The development of new antibacterial coatings and surface modification strategies to prevent unfavorable bacterial attachment are considered as excellent infection preventative measures (Ref 193). These strategies prevent microbe infections through the addition of self-sanitizing properties to the surfaces in the form of thin coatings (Ref 194, 195), improvement of biocidal-release rate using surface modification methods (Ref 176, 196, 197), reduction of the microbial surface attachment using surface topography modification methods (Ref 198-200), and contact killing of bacteria using nanostructured surfaces (Ref 201-203). When designing antibacterial surfaces (Ref 204, 205), aside from their effectiveness in biofilm formation inhibition, other considerations should also be considered. For example, continuous release of metallic biocidal products to the environment may contribute to the development of microbial strain resistance to these biocidal agents (Ref 206). Other parameters such as surface durability (Ref 207) and surface cleaning strategies (Ref 208) are factors that need precise assessment before introducing the antibacterial surfaces to the market.

Recent advances in surface modification techniques, such as CS, chemical vapor deposition, plasma treatment, along with progress in powder metallurgy have enabled the fabrication of composite materials with successful embedment of antibacterial agent such as Cu, Pb, Ni, Ag, and Zn in ceramic and polymer matrix (Ref 193). It was shown that embedment of copper particles into thermoplastic polymers can promote surface performance against fouling organisms (Ref 209). Using copper nanoparticles (Cu-NPs) may also enhance surface antibacterial properties beyond what is normally observed in bulk copper (Ref 210). Attractive antibacterial properties of zinc oxide nanoparticles (ZnO-NPs) have been a subject of interest worldwide (Ref 211). The enhanced antibacterial properties of Cu-NPs and ZnO-NPs can be attributed to the increased specific surface area as the reduced particle size leads to enhanced particle surface reactivity (Ref 211).

The antibacterial properties of silver, nickel, zinc, and copper substituted hydroxyapatite composite coatings using CS were investigated and showed promising antibacterial performance (Ref 212). The solid-state nature of CS allows deposition of mixed materials with different inherent antibacterial properties, which may boost the surface antibacterial effectiveness to a superior level. For example, materials, such as Cu and titanium dioxide (TiO_2_) are effective antibacterial agents with different killing mechanisms; Cu through contact killing in presence visible light and TiO_2_ through reactive oxygen species under ultraviolet light (Ref 213, 214). CS can be used to fabricate a surface of a mixture of these two materials (Ref 215), which has the potential to be a superior antibacterial surface in the field of visible light (Ref 212).

CS also allows the deposition of nanoparticle antibacterial agents, such as Al, Cu, Ti (Ref 216-218). For example, graphene oxide (RGO) silver-nanoparticles (Ag-NPs) aluminum composite powder deposited on a mild steel plate using CS demonstrated high antibacterial activity and improved mechanical properties with the RGO/Ag-NPs powder structure retained in the coating (Ref 219). Another example is the deposition of ZnO-NPs mixed with Cu on stainless steel substrates to achieve antifouling coating for marine application. The results demonstrated that hydrophobic ZnO/Cu coating was able to inhibit the attachment of Caloplaca marina effectively (Ref 217)). In another study, ZnO/Ti powder were blended mechanically in a ball-mill and were deposited on Al 6061 substrates. Different powder mixture combinations were studied, and the results showed a significant antibacterial effectiveness against E. coli (Ref 218). In a similar study, the antibacterial activity of mixed ZnO nanopowder and aluminum powder on glass substrates were investigated (Ref 216). It was observed that the antibacterial activity increased with increasing ZnO nanopowder concentration in the cold sprayed coating (Ref 216). However, the increase in ZnO-NPs results in low deposition efficiency and coating adhesion strength. This is also the main challenge when dealing with the deposition of ceramic powders such as ZnO and TiO_2_ particles.

One way to address these challenges is by designing powders that allow the deposition of ceramic powders and nanoparticles while keeping the ratio of cementing metal binder phase proportionally minimal. This can be achieved by careful selection of matrix and a reinforcement phase, as well as the powder production methods (Ref 220). Powder production methods such as ball-milling, agglomerating and sintering, spray drying, wet chemically synthesizing and agglomerating, or combination of these powder preparation methods can be adapted to create powder with desired mechanical, microstructural, and antiviral properties. Once optimal powders are produced, CS can potentially be used for the fabrication of any desired powder combination at sites where antibacterial coatings are needed or repair are required. Examples can be the in-situ deposition of ZnO/CuO nanocomposite. ZnO/CuO nanocomposite synthesized by a chemical Co-precipitation approach exhibited far superior antibacterial activity as compared to ZnO (Ref 221). Producing surfaces with nanopattern roughness directly using powders with nano-features is not far reached using CS. An example might be producing ZnO nanopattern coating directly by spraying nano featured ZnO powders.

### CS Potential for Hydrophobic-Icephobic Coatings

Ice accumulation can result in the failure of wind turbines (Ref 222, 223), power networks (Ref 224, 225), aircrafts (Ref 224, 226, 227), and many other systems exposed to atmospheric icing conditions. The development of a passive anti-icing (icephobic) solution has been a topic of interest to prevent ice accumulation on these critical structures, however, their application is not widespread at this time (Ref 226). A surface is deemed icephobic if it can prevent or reduce the accumulation of ice through low adhesion, superhydrophobic behavior at low temperatures, or prevention of ice nucleation (Ref 224, 228, 229). Surface icephobicity is achieved through the combination of physical surface characteristics, such as surface topography, and through the surface chemical properties of the selected materials (Ref 230). Smooth coatings are commonly used as ice release surfaces since ice adhesion is influenced by surface roughness as the ice can mechanically interlock with asperities (Ref 231-233). However, reports of textured surfaces, such as superhydrophobic surfaces (SHS), have also been explored extensively for their use in icing environments. Their contact area with water droplets is low, and they have the ability to rebound impacting supercooled water droplets before they freeze (Ref 233-235). Low surface energy has become an important requirement for anti-icing surfaces to create a high free energy barrier, leading to increased hydrophobicity and ice nucleation time (Ref 224, 229). Low surface energy materials, such as polyolefin plastics and quasicrystal materials (QC), have been known to slow down ice nucleation and reduce adhesion (Ref 228, 235, 236). A large contribution to ice adhesion is also attributed to electrostatic forces, which refers to imposed surface charges by the ice which are mirrored by the substrate, resulting in a chemical bond (Ref 235, 237). Surface charges are mirrored very easily in metallic materials, however insulators with low dielectric constants, such as PTFE, can significantly decrease the electrostatic bonds, and thus, reduce ice adhesion (Ref 235, 237). Icephobic surfaces are increasingly being produced by thermal spray technologies. For instance, Koivuluoto et al. have produced polyethylene-based coatings, solid lubricant coatings, and slippery liquid-infused porous surface (SLIPS) coatings using the flame spray process (Ref 238-240). Mora et al. have demonstrated icephobic properties of QC coatings that were produced using the high-velocity oxy-fuel process (Ref 236). However, these processes rely on elevated processing temperatures which can negatively impact the feedstock and deposited material.

**Fig. 21:**
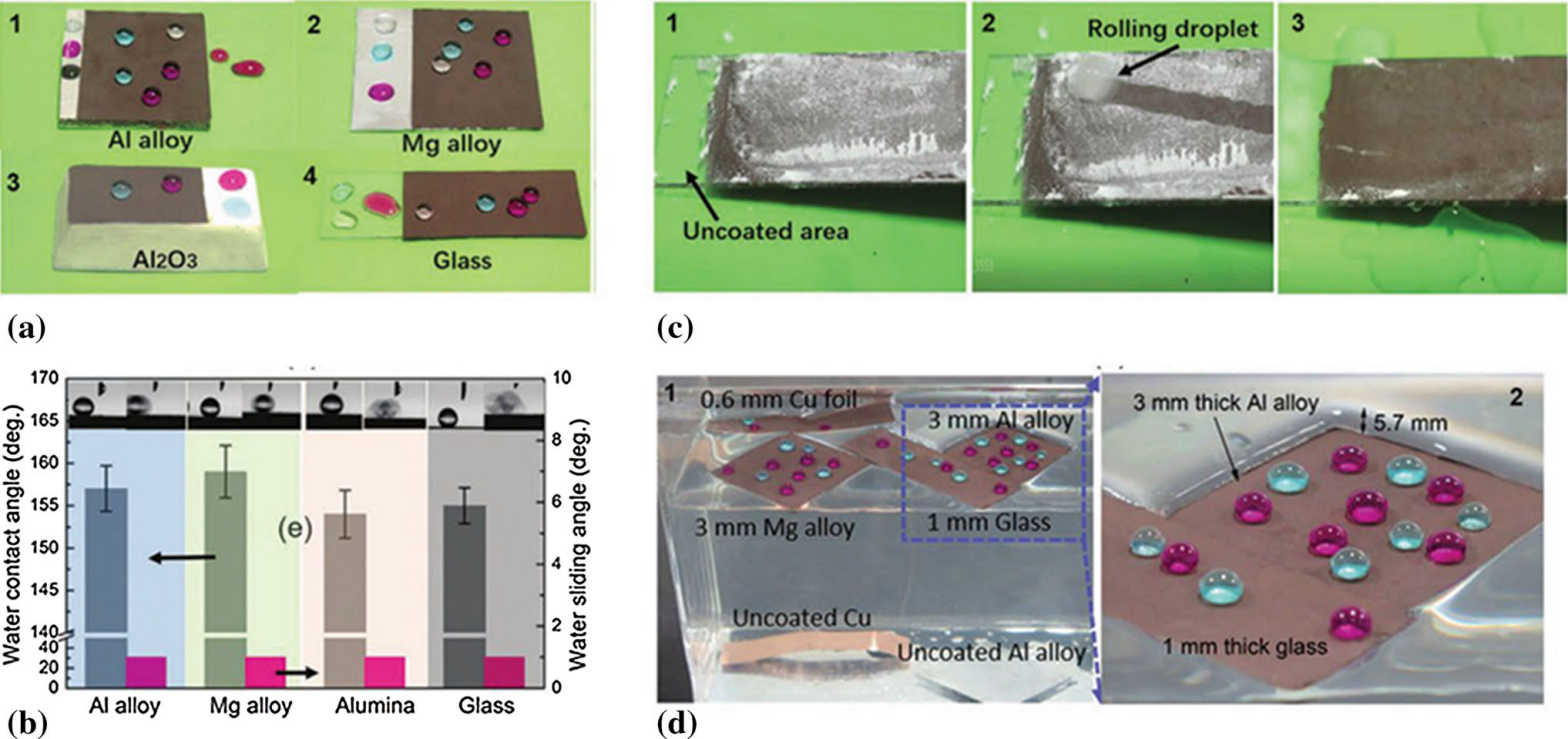
The water repellency of cold sprayed Cu-based superhydrophobic coating: (a) the superhydrophobic performance on different substrate materials including (a1) Al alloy, (a2) Mg Alloy, (a3) Al2O3 ceramic and (a4) optical glass; (b) the results of water contact angle and water sliding angle on different substrates; (c) self-cleaning test result by using sticky starch powders; (d) the enhanced floating ability of different plates with superhydrophobic coating (Ref 244)

CS has the potential to produce three types of icephobic surfaces: smooth surfaces, textured SHS, and porous surfaces to be used as SLIPS. For example, thin and dense cold sprayed coatings can be grinded or polished to produce smooth surfaces. Surfaces created with CS could also be engineered to have the required topography to be superhydrophobic using a masking technique (Ref 169, 241). Slippery Liquid-Infused Porous Surfaces (SLIPS), requiring a very porous coating for the infusion of a liquid water repellant to reduce ice adhesion, can also be potentially produced by CS with polymer feedstock as well as other matrix materials with optimized spray conditions to achieve the required porosity (Ref 240, 242). The production of solid lubricant coatings is not a new theme for CS, however, they have yet to be implemented as icephobic surfaces (Ref 243). Figure 21 shows a type of Cu-based superhydrophobic coating fabricated by cold spray followed by flame oxidation. The excellent superhydrophobic performance is attributed to the coral-reef-like hierarchical flexible architectures of feedstock powder and the wear-resistant porous oxide surface layer provided by flame oxidation process.

For the deposition of polymers in cold spray, the low melting temperature, irregular morphology, limited flowability, and absence of metallic bonding are all hurdles to overcome (Ref 245-248). Furthermore, the low durability of polymer/elastomer coatings is also a drawback (not specific to CS) that needs to be addressed. Icephobic elastomer and polymer surfaces currently struggle to provide durable surfaces that can sustain multiple icing and de-icing cycles and provide UV protection, erosion protection, and others (Ref 226, 233, 249). Conversely, for materials like QCs, durability is not the issue but depositing such brittle particles at low temperatures can prove to be difficult (Ref 250).

Achieving the full potential of CS in creating icephobic coatings lies in the resolution of the aforementioned challenges. To overcome the brittleness of QCs, the development of powerful and reliable powder preheaters may be required to reach a more ductile state without relying on extreme spray parameters that could lead to substrate damage, but this has yet to be studied directly with CS (Ref 250, 251). Obtaining more durable polymeric coatings remains a challenge that every coating deposition method is currently investigating, although CS could provide a new perspective. Depositing low melting temperature feedstock, such as polymers, remains a challenge for the CS process, however different avenues are being explored such as new nozzle designs (Ref 247) and substrate preheating to increase adhesion (Ref 245). Glass structures such as windshields, windows, and solar panels would benefit from icephobic properties, with transparency a firm constraint to their overall function. It is possible to envision polymer feedstock being re-engineered and tailored to the CS process as well as developing new CS spray windows for these materials to achieve a level of transparency, or translucency. The use of CS could be especially advantageous when applying such coatings on mobile phones or automobiles, where the large production volume requires fast application and little post-processing. Other innovations in icephobic coatings include the use of photothermal materials to generate heat from solar radiation (Ref 252, 253). The photothermal effects introduce a sustainable and passive solution to achieve icephobicity without the need for any external energy source. Photothermal materials that should be explored using CS are metallic nanostructures, inorganic semiconductor, and polymeric materials. Self-healing, durable icephobic polymeric surfaces could prove useful (Ref 254) in many applications. In addition, repairing these coatings in the field could be resolved by using CS. The portability of CS systems could reduce the need for spare parts and alternatively promote applying reparation coatings if needed.

### Photocatalytic Materials/Coatings

Photocatalytic materials have been widely explored and used for purifying and sanitizing water and air (Ref 255-257). These materials form conduction band electrons (e) and valence band holes (h^+^) upon light excitation that react with water and oxygen and form reactive oxidizing species such as •O_2_^-^, •OH, and •HO_2_. These species react with impurities, bacteria, and viruses to accelerate their decomposition (Ref 255-259). TiO_2_ is a photocatalytic material that has many advantages such as high chemical stability, nontoxicity, high reactivity, good durability, and cost effectiveness, and therefore, it has been extensively studied for photocatalytic applications (Ref 256-263). Two common TiO_2_ phases are anatase and rutile. Anatase is categorized as more reactive due to its high degree of lattice oxygen anion (${\mathrm{O}}^{2-}$) displacement, while rutile is the most stable form of TiO_2_. Studies have shown higher reactivity in anatase-rutile mixtures due to the presence of heterogeneous interphase junctions (Ref 263-265). This combination enhances the redox reaction properties of the catalyst and leads to an increase in carrier separation efficiency at the interface, thus allowing for overall higher reactivity in the junction than either single phase material (Ref 266). Various processes have been used to produce photocatalytic TiO_2_ coatings. Traditional methods include physical/chemical vapor deposition and sol-gel techniques (Ref 267, 268). Thermal spray processes have been explored due to their fast deposition rates, flexibility on powder and substrate selection, and capability of coating large surfaces (Ref 262, 269-272). A main challenge in thermal spray deposition of TiO_2_ coatings is maintaining the heterojunctions because high process temperatures lead to grain growth and anatase-to-rutile transitions (Ref 273, 274).

CS, due to its low process temperature, has been shown as an alternative process for producing photocatalytic nanostructured TiO_2_ coatings (Ref 258, 275-277). Research shows that spraying at lower temperatures prevents phase or surface morphology changes in the titanium dioxide, and therefore, the anatase and rutile phases of the feedstock powder can be retained in the TiO_2_ coatings (Ref 258, 259, 277, 278). However, being a ceramic material, deposition of TiO_2_ powders using CS remains challenging as plastic deformation is typically required to promote substrate-particle and interparticle bonding. Due to its lack of plastic deformation, TiO_2_ powder is more likely to shatter and/or embed in the substrate. This makes it difficult to build up TiO_2_ coatings. Fortunately, photocatalytic reaction is a surface phenomenon, and a thin layer of TiO_2_ coverage could perform well in photocatalytic tests (Ref 258, 259), thus facilitating the implementation and use of CS deposition.

A potential way to produce successful CS TiO_2_ coatings is through the use of feedstock powders with a pure titanium core and a TiO_2_ shell. Such powders can be produced by chemical treatment of pure titanium (Ti) using solutions based on hydrogen peroxide (${\mathrm{H}}_{2}{\mathrm{O}}_{2}$) (Ref 262, 279-282) or sodium hydroxide (NaOH) (Ref 283-286). This can develop an oxide layer surrounding the pure Ti particle that consists of the desired nanostructured heterojunctions of anatase and rutile. It is foreseen that during CS application the pure Ti core would deform while the TiO_2_ shell may be shattered and trapped between Ti particles as well as on the surface. As CS typically maintains or even refines the powder’s crystalline structure (Ref 258, 259, 277, 278), the TiO_2_ nanostructures and heterogenous junctions grown on the Ti particles from the chemical treatments could potentially be maintained in the resulting Ti/TiO_2_ coatings. Overall, CS applications of modified pure Ti having a TiO_2_ oxide layer possess the potential of successful deposition that can be applied to various industries, particularly on-site coating deposition and repair in situations where components cannot or preferably not be dismantled. Researchers aim to increase the reactivity of titanium dioxide to a larger electromagnetic spectrum, as it is currently reactive to about 3-5% of the entire sunlight spectrum (Ref 255, 265). Theoretical research and experiments are being examined in advancing visible light activation, understanding the origin of visible light activity, and the electronic structures of various visible light active titanium dioxide photocatalysts. This includes identifying dopants, metal and non-metal, that can improve visible light absorption and electron-hole separation (Ref 255, 287-290). As well as dopants, modifying titanium dioxide with carbonaceous materials and coupling it with other metal semiconductors is researched for activation under visible light. Alternatively, utilizing transition metals with titanium dioxide can narrow the band gap and cause a red shift into of the optical absorption edge into the visible region (Ref 287–290). Many avenues of research are being explored into increasing the sensitivity and reactivity of titanium dioxide such as chemically altering its composition, metal and non-metal ion doping, combining with other materials and introducing both organic and inorganic coatings. If these methods are proven successful, then this could mean subsequent involvement with CS applications that work in conjunction to optimize the heterojunctions and maintain microstructure. Therefore, further research endeavors for the CS application of titanium dioxide would include continuing optimizing applications and maximizing the photocatalytic properties to react under visible light as opposed to just ultraviolet light.

### Power Generation & High Temperature Materials

#### MCrAlYs for Bond Coats and Thermal Barrier Coatings

MCrAlYs, where M stands for Ni and/or Co, are common bond coat materials for thermal barrier coatings (TBCs) used in gas turbine engines (Ref 291–295). Their composition is designed to preferentially develop a dense, stable, and continuous protective α-alumina oxide scale upon high temperature exposure. This thermally grown oxide (TGO) layer provides enhanced resistance to high temperature oxidation during operation. Studies have shown that better control of the bond coat microstructure and oxidation behaviour is highly beneficial to the performance and thermal cycling durability of TBCs (Ref 286–299). MCrAlY bond coats are typically manufactured by thermal spray methods such as APS, LPPS, or HVOF (Ref 300–302). All these processes involve significant heating of the feedstock material, which causes full or partial melting of sprayed particles and often results in detrimental microstructural changes in the coatings (Ref 288, 303).

Given its relatively low process temperature and general suitability for metallic materials, CS has also been widely investigated as an alternative manufacturing method for bond coats. Dense MCrAlY coatings with favorable microstructures can be successfully produced using CS, leading to improved high temperature oxidation resistance in comparison with other thermal spray bond coats (Ref 303–307). Furthermore, CS is capable of depositing nanocrystalline feedstock powders and retaining their nanocrystalline microstructures into the resulting coatings (Ref 308–312). In some cases, the extensive plastic deformation inherent to CS can cause significant localized grain refinement in MCrAlY materials and result in in-situ nanocrystallization of its microstructure (Ref 313–315). Nanocrystalline bond coats were shown to exhibit improved oxidation behaviour as a result of the finer grain structure and increased grain boundary area, which promoted aluminum diffusion and provided a greater network of nucleation sites for the initial formation of the desirable α-alumina oxide scale (Ref 306, 316–319).

To successfully integrate CS bond coats into industrial TBC production, economical challenges will first need to be overcome. One prominent challenge is the high production costs: MCrAlY powders typically require high particle impact velocity to be deposited using CS (Ref 320), often necessitating the use of expensive helium as the process gas, which is also classified as a sensitive non-renewable resource. Although helium recycling systems are effective in reducing gas consumption costs (Ref 47, 321), they also represent a large capital investment and therefore have had limited use. Alternatively, the use of a cost-effective gas, such as nitrogen, often results in low particle impact velocities and consequently lower deposition efficiencies (DE). This in turn can lead to large amounts of wasted powder as well as longer spray times, thereby increasing material, gas and labor costs. It is envisioned that a variety of different approaches could be considered in order to make CS economically competitive with other manufacturing methods. Many of these potential solutions could explore opportunities to improve DE when using nitrogen as the process gas, such as further elevating gas stagnation parameters, improving particle size distribution control (Ref 322) along with nozzle design optimization, and achieving higher particle impact temperatures via enhanced powder preheating. In-situ substrate heating and surface conditioning, such as in laser-assisted CS (Ref 85, 323) and induction heating CS (Ref 324, 325), have shown promise in increasing DE with other challenging materials and should also be considered for MCrAlYs. Another approach is gas mixing, where nitrogen and helium gases are blended to an optimal ratio in order to minimize process costs (Ref 326, 327). Efforts have also been made to investigate the viability of powder recycling (Ref 169, 328), and although more development work is required, preliminary findings appear promising.

With the development of columnar topcoat microstructures using suspension plasma spray processes (Ref 329, 330), exciting future opportunities exist with the evaluation and optimization of TBC systems featuring such a topcoat and an underlying CS MCrAlY bond coat. This could potentially provide a similar microstructure and performance as that of TBCs with an EB-PVD topcoat and a platinum-modified diffusion aluminide or a LPPS MCrAlY bond coat, but at a significantly lower production cost. In addition, as a promising additive manufacturing process, CS could potentially be used to manufacture entire gas turbine components with an integrated MCrAlY bond coat in a single setting. Such a process could allow for functionally graded compositions near the substrate/bond coat interface to better accommodate typical issues such as element inter-diffusion and undesirable microstructure evolution. It might also be possible to include fine alumina particles in the sprayed powder in order to embed them in the upper layers of the bond coat and investigate if improvements to the oxidation behavior and longevity of the TBC in thermal cycling may be achieved. Finally, CS as an additive manufacturing approach could also allow for more sophisticated internal coolant channel networks for enhanced cooling effectiveness and component durability.

#### Temperature Resistant Materials

Coatings to be used in harsh environments and with high mechanical properties at elevated temperatures are of great importance for the advancement of power generation technologies, such as in pressure tubes in thermal and nuclear power plants, in chemical factories, in the oil gas generation industry, and inside boilers where waste and biomass are used (Ref 331). Cost effective and thick coatings of nickel-based superalloys (Ref 332, 333), steel and stainless steel alloys (Ref 334-336), titanium alloys (Ref 337, 338), tantalum (Ref 339, 340), and niobium (Ref 341, 342) have been produced using several thermal spray processes, such as HVOF, HVAF, APS,WAS, and TWAS (Ref 333, 334, 336, 337, 343, 344). They are usually characterized by a high-oxide content (Ref 334, 336, 339, 345), high porosity levels (Ref 334, 337, 340, 344-346), and micro-cracks (Ref 343, 345, 346). These defects are to be avoided as they can promote premature failure of the coating. Substrate deformation may also occur affecting the integrity of the material to be coated (Ref 345, 346).

The CS process has emerged as a possible solution for the production of coatings for the power generation industry (Ref 333, 339, 343, 347, 348). Due to their high strength and low ductility, CS deposition of materials such as Inconel 625, Inconel 718, tantalum, Ti-6Al-4V, and stainless steel alloys require the use of process gas temperatures ranging up to 1000 °C and pressures up to 5 MPa, or the use of helium as process gas instead of nitrogen (Ref 157, 333, 339, 340, 347-352). High gas temperatures are used to increase the gas velocity and to raise particle impact temperature (Ref 340, 348, 350, 352). In some cases, an optimized particle size distribution has been required to maximize the coating quality (Ref 340, 348, 350, 352). In others, adding to the feedstock powder a hard secondary powder material has been needed to obtain a high coating quality (Ref 105, 353–355). This latter approach reduces the DE of the material of interest and results in deposition of the secondary powder, leading to coating contamination that can be detrimental (Ref 105, 353–355). Problems such as nozzle clogging (Ref 157, 347, 348), substrate bending (Ref 343, 356), and the cost of using helium (Ref 157, 327, 333, 351) have made the adoption of the CS process more challenging for the industry.

The laser-assisted CS process has emerged as a potential solution to the current problems faced for the deposition of high-strength materials (Ref 85, 87, 323, 348, 356, 357). The laser softens the coating layers promoting higher deposition rates and denser coatings (Ref 85, 87, 323, 356, 358). The main drawback of this assisted process is that the heating pattern is limited to the spot size of the laser. In addition to that, tighter safety guidelines must be enforced due to the potential hazard of operating a laser system (Ref 87, 357). Another potential solution proposed is the induction heating cold spray (IHCS) process (Ref 324, 359). In this process, the induction heating is used to preheat the substrate and the coating during the deposition process. Similar to the laser-assisted process, the IHCS also softens the coating layers for enhanced deposition properties while coating heating is not limited to the spot size of the laser, and thus, a more uniform temperatures can be achieved (Ref 324, 359). Heat treating the powder prior to its deposition has been explored, as microstructures that favor plastic deformation can be obtained (Ref 360-363). This approach will possibly help produce coatings of materials that have not yet been investigated.

### High Entropy Alloys (HEAs)

#### Cold Spraying of HEAs

The development of high entropy alloys (HEAs) has brought a new realm of research within the material science field (Ref 364). HEAs are defined as a solid solution alloy of four or more principal elements in equiatomic or near-equi-atomic ratios, and they typically exhibit a single- or dual-phase structure. HEAs provide an excellent combination of strength and ductility, outstanding irradiation resistance, high corrosion and oxidation resistance, and excellent wear resistance when compared to conventional alloys. In addition to the composition, the properties of HEAs also depend on the preparation, manufacturing, and final consolidation route utilized. Solid-state mechanical alloying (MA) or powder atomising process are now commonly used to produce pre-alloyed HEA material. Deposition of HEA powder into coatings using laser and plasma cladding, plasma spray and magnetron sputtering have been performed. However, the high process temperatures generating melting increases the possibility of dilution with the substrate, segregation, and producing undesired precipitates and intermetallics (Ref 365, 366).

**Fig. 22:**
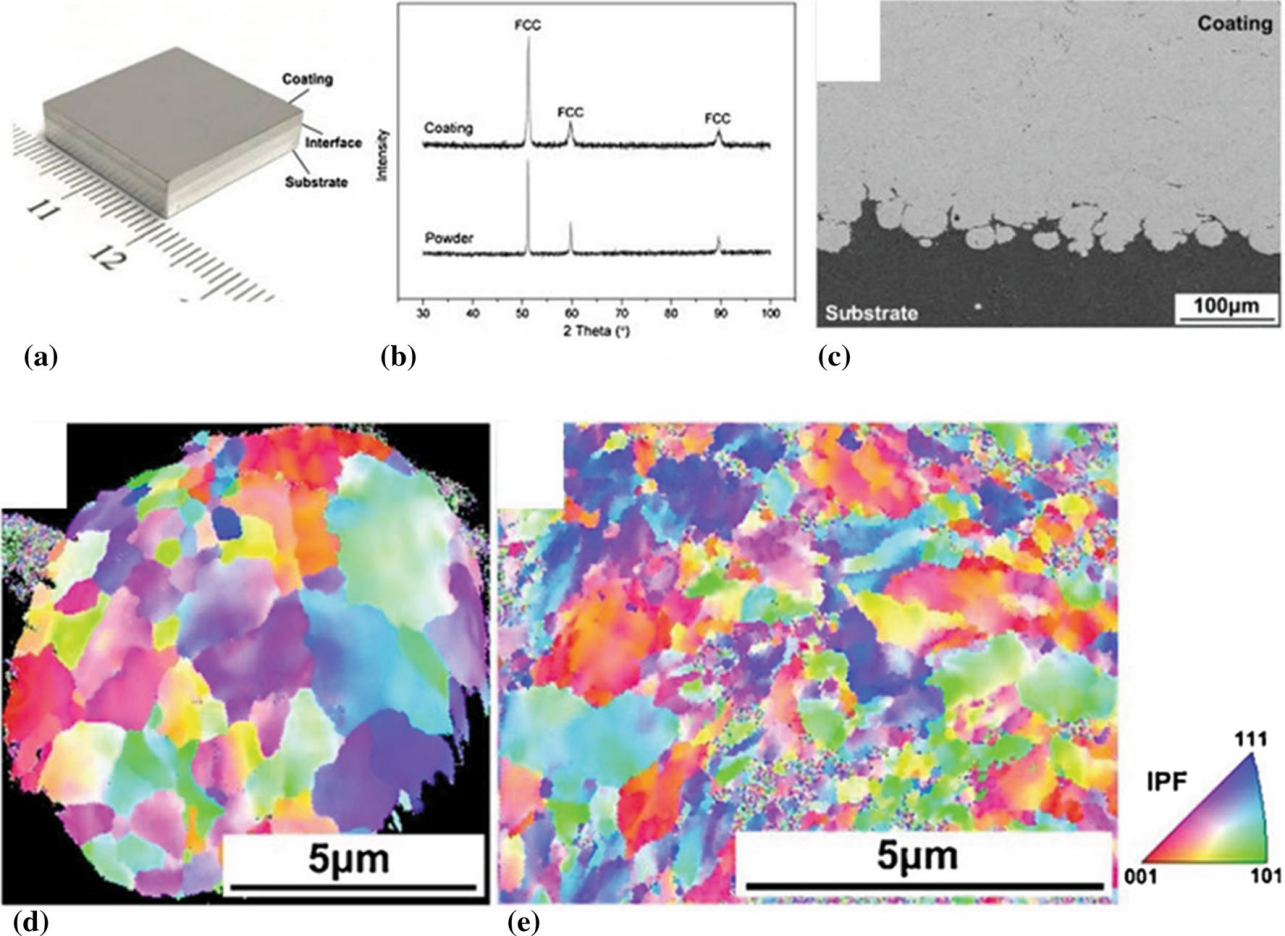
Cold sprayed FeCoNiCrMn HEA coating: (a) image of the HEA coating on the substrate, (b) XRD spectra of the HEA powder and coating, (c) cross-sectional SEM image of the HEA coating, EBSD IPF maps of (d) a single HEA particle, and (e) the cold sprayed HEA coating (Ref 370)

**Fig. 23:**
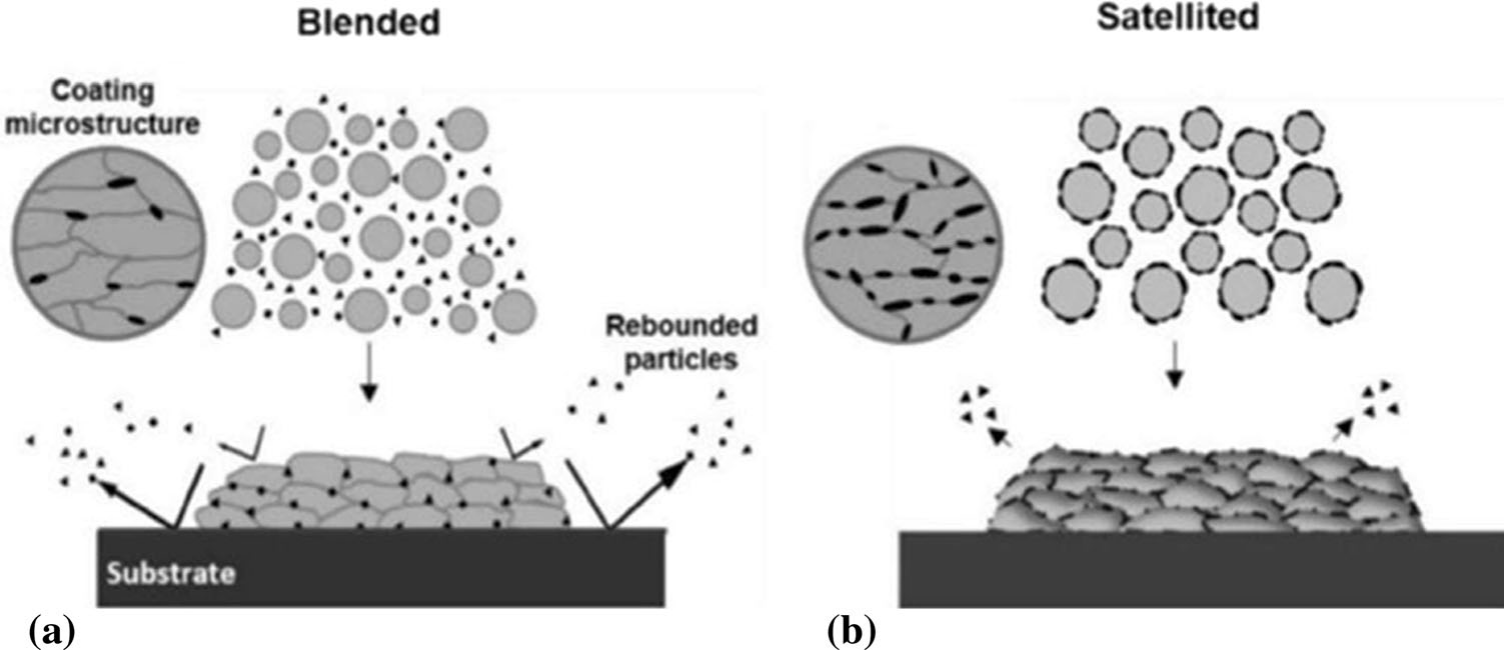
Schematic illustration of two typical cold sprayed MMC coating formation mechanisms using: (a) blended feedstock and (b) satellited feedstock (Ref 384)

To avoid this drawback, CS deposition of HEA has been explored and materials such as AlCoCrFeNi, FeCoNiCrMn, AlFeNiCoCrTi, and CrFeNiMn have been studied (Ref 365-370). The authors’ group is the very first research team worldwide that confirms the feasibility of cold spraying to prepare HEA deposits. As shown in Fig. 22, the cold sprayed FeCoNiCrMn HEA deposit presents a dense structure. In addition, the deposit not only retains the initial phase composition and structure of the HEA feedstock powders, but also increases the hardness through severe plastic deformation, i.e., grain refinement and work hardening. The CS process not only retains the initial phase composition and structure of the HEA powder, but also increases the hardness through severe plastic deformation, i.e., grain refinement and work hardening. A recent research study has shown that severe plastic deformation can enhance the impact and energy absorption of specific HEAs by formation of hierarchical microstructure including stacking faults, twins, grain refinement, and amorphization (Ref 371). Therefore, CS coatings which typically are formed under a high strain rate, cold, and severe plastic deformation (Ref 372) could enhance the resistance of the alloy in extreme loading conditions and the energy absorption capacity of HEA coatings/parts. CS HEA coatings have shown increased microhardness (Ref 366), oxidation resistance at high temperature (Ref 373) and lower wear rate (Ref 370) compared to conventional alloys. However, the extensive grain boundary network and presence of porosity within the CS coating has shown an increase in internal oxidation otherwise not detected in bulk HEA material (Ref 373). This is related to the fact that CS deposition of HEAs is challenging (Ref 367) mainly due to their excellent work hardening and low thermal softening (Ref 367). Hence, optimization of CS process parameters in the deposition of HEA should be the near-term focus. CS HEA coatings usually were sprayed with He as the process gas to achieve high impact velocity and enhance CS deposition (Ref 365-370) while He is an expensive and scarce source. The perspective in the sustainable development of CS of HEAs and enhancement of deposition is to use hybrid CS deposition methods including laser-assisted cold spray and particle/substrate preheating and using N_2_ as propelling gas (Fig. 23).

The emergence of HEA materials and their unique functional properties has attracted a variety of new industrial applications. The following sections focus on the energy and environmental fields as they are increasingly the focus of global research studies.

#### Energy Storage, Sensing, and Radiation Protection

Metals with BCC structures and containing Laves phases have shown high reactivity with hydrogen at room temperature and are considered as promising hydrogen storage materials for stationary applications (Ref 374). It has been shown that the high entropy feature of HEA materials promotes the formation of Laves phases and BCC structures (Ref 375). The ability of the CS process to retain the initial powder structure/composition after deposition enables the production of materials for hydrogen storage. Another potential application for the CS process is the deposition of Pd-rich HEA materials, which are hydrogen-sensitive materials with high sensitivity, fast response, good stability and recyclability for H_2_ sensors capable of detecting H_2_ leakage (Ref 376).

The AlCoCrFeNi HEA material volumetric capacitance and cycling stability increases with the presence of nanoporosity and refined microstructure (Ref 377). Since the CS process generates recrystallization and particle-to-particle interface porosity, it can be used to deposit supercapacitor electrodes with high electrochemical property.

The use of structural materials resistant to radiation damage, i.e., harsh environments with high temperature and radiation dose, is a great interest as austenitic steels, nickel-based superalloys, and zirconium alloys can only withstand up to 10 dpa irradiation. HEA materials under irradiation have shown significantly lower material expansion and increased effective absorption bandwidth than that of traditional materials (Ref 376). Furthermore, owing to their superior mechanical/chemical properties and apparent resistance to radiation, refractory HEAs have been proposed as promising candidates for advanced nuclear fusion/fission reactor applications (Ref 378). Additionally, various HEA (FeCoNiCrAl, FeCoNiCuAl, FeCoNiSi_0.4_Al_0.4_, and AlCoCrFeNi) successfully deposited through CS have been tested and their ability to absorb electromagnetic wave (EMW) demonstrated. Hence, with increasing development of wireless technology and electronic devices and need for hard radiation resistant coatings, the CS process future is becoming increasingly broad.

### Metal Matrix Composites

The advantage of the CS process in the production of metal matrix composite (MMC) material is the low working temperature, which eliminates oxidation and possible matrix-reinforcement (ceramic, metallic, or intermetallic) interfacial reactions, thus preserving the feedstock material microstructure (Ref 379). CS deposition of MMC can be divided into two main categories based on the nature of the reinforcement materials: metal-metal MMCs and ceramic-metal MMCs. In metal-metal MMCs, where each component usually can be deposited individually by CS, the interaction of dissimilar materials changes and usually improves the DE of the mixed powder from that of single component deposition (Ref 380). In ceramic-metal MMCs, the reinforcement material (hard particles such as Al_2_O_3_) added in small amounts can increase the DE and decrease the coating porosity (Ref 381). Generally, maximum deposition efficiency is reached with a volume fraction of hard reinforcement powder in the range of 20-40% (Ref 381). On the other hand, some other ceramic phases such as hBN and MoS_2_, which have abradable functions, decrease the DE to some extent (Ref 382).CS of two former groups of MMCs were well studied and matured while the latter is still in the way of improvement by optimization of CS processes. Recently, pulsed-gas dynamic spray of Cu-hBN MMCs offered a promising development path for CS of abradable coatings (Ref 382). MMCs have shown a broad potential in numerous applications, and with recent advances in CS additive manufacturing, bulk free-standing MMC CS deposits are gaining more interest (Ref 254, 383). However, more research must be undertaken to understand the load-bearing behavior of composite coatings, i.e., state of strain and stress, at the reinforcement interface, which can vary from measured macroscopic values and theoretically predicted magnitudes.

## Conclusions

The current paper summarizes the major milestones of the cold spay technique that mainly carved its evolution to its modern form, following several exploratory and developmental investigations. The highlights are the following:

- Modern commercial CS systems can achieve pressure over 60 bar at the nozzle inlet, with a preheating gas temperature that exceeds 1000 °C. These levels have more than tripled since the process early days. This progress has enabled CS processing of materials that were initially challenging to deposit (such as WC-Co) with relative ease.
- The greatest nemesis of CS has been its inherently high processing cost when helium is used. The need of being able to apply this process for industrial applications at a low cost has resulted in the development of CS variants such as the laser cold spray and the pulsed cold spray, using nitrogen as a propellant gas. Over the years, the expectations of the scientific community from CS were higher than the process that could technically achieve (especially in terms of applications), resulting in a declining level of interest in the 2010-2015 period.
- Following the discovery of more novel applications (such as for the repair of high value parts in the military sector), CS has gained new interest that is nowadays progressively (not abruptly) increasing. The process has been widely applied for copper (mainly thermal) and aluminum (mainly repair) deposition.
- The outbreak of Covid-19 has triggered innovation in surface sanitation, and copper deposited via cold spray was proven very efficient for viral inactivation as an example. This may well lead to high volume applications, especially when considering the deposition over polymer surfaces that is a relatively complex operation when using alternative processes. Proven potential for deposition of thermal barrier coatings, high temperature materials, high entropy alloys, energy storage/radiation protection materials, and metal matrix composites have also been identified. It is unclear at this stage the precise role and impact CS will have in these areas, however these are likely those who will benefit the most and tangible applications are starting to emerge. An example is the use of copper-CS in Canada to seal radioactive waste containers. In summary, the developmental journey of this process has been quite extraordinary and exciting.

Currently, CS equipment is available commercially worldwide. While an exponential explosion of possible CS applications is no longer anticipated, the most recent advances will consolidate the existing knowledge and experience and will form the basis for new areas to explore. For instance, as new powder metallurgy routes are investigated, new powders are specifically designed for CS processing. Therefore, the likelihood of being disruptive will be high, also in areas where other thermal spray processes still dominate.

## Footnotes

1 Ford Motor Company, General Motors, General Electric Aircraft Engines, and Pratt & Whitney Division of United Technologies, General Electric Aircraft Engines, Tubalcain Company, and Flame Spray Industries.
